# Insights into myalgic encephalomyelitis/chronic fatigue syndrome phenotypes through comprehensive metabolomics

**DOI:** 10.1038/s41598-018-28477-9

**Published:** 2018-07-03

**Authors:** Dorottya Nagy-Szakal, Dinesh K. Barupal, Bohyun Lee, Xiaoyu Che, Brent L. Williams, Ellie J. R. Kahn, Joy E. Ukaigwe, Lucinda Bateman, Nancy G. Klimas, Anthony L. Komaroff, Susan Levine, Jose G. Montoya, Daniel L. Peterson, Bruce Levin, Mady Hornig, Oliver Fiehn, W. Ian Lipkin

**Affiliations:** 1 Center for Infection and Immunity, Columbia University Mailman School of Public Health, New York, NY 10032 USA; 2 UC Davis Genome Center - Metabolomics, University of California, Davis, CA, 95616 CA USA; 3 Bateman Horne Center, Salt Lake City, UT 84102 USA; 4 Institute for Neuro Immune Medicine, College of Osteopathic Medicine, Nova Southeastern University, Fort Lauderdale, FL 33314 USA; 5 Miami VA Medical Center, Miami, FL 33125 USA; 6 Harvard Medical School, Brigham and Women’s Hospital, Boston, MA 02115 USA; 7 Levine Clinic, New York, NY 10021 USA; 8 Stanford University, Palo Alto, CA 94305 USA; 9 Sierra Internal Medicine at Incline Village, Incline Village, NV 89451 USA; 10 Department of Biostatistics, Columbia University Mailman School of Public Health, New York, NY 10032 USA

## Abstract

The pathogenesis of ME/CFS, a disease characterized by fatigue, cognitive dysfunction, sleep disturbances, orthostatic intolerance, fever, irritable bowel syndrome (IBS), and lymphadenopathy, is poorly understood. We report biomarker discovery and topological analysis of plasma metabolomic, fecal bacterial metagenomic, and clinical data from 50 ME/CFS patients and 50 healthy controls. We confirm reports of altered plasma levels of choline, carnitine and complex lipid metabolites and demonstrate that patients with ME/CFS and IBS have increased plasma levels of ceramide. Integration of fecal metagenomic and plasma metabolomic data resulted in a stronger predictive model of ME/CFS (cross-validated AUC = 0.836) than either metagenomic (cross-validated AUC = 0.745) or metabolomic (cross-validated AUC = 0.820) analysis alone. Our findings may provide insights into the pathogenesis of ME/CFS and its subtypes and suggest pathways for the development of diagnostic and therapeutic strategies.

## Introduction

Myalgic encephalomyelitis/chronic fatigue syndrome (ME/CFS) is a disorder of more than six months duration comprising unexplained fatigue, post-exertional malaise, unrefreshing sleep and either cognitive dysfunction or orthostatic intolerance^1^. Between 800,000 and 2.5 million people in the United States alone are estimated to have ME/CFS^1^. There is no approved laboratory diagnostic test; hence, the diagnosis is based on history, physical examination and exclusion of other disorders^1^. Patients with ME/CFS frequently report a prodrome consistent with infection that includes a sore throat and cervical lymphadenopathy^1^. An estimated 35% to 90% of patients have irritable bowel syndrome (IBS)^2–4^, compared to 10–20% of the general population^5,6^.

Metabolomic studies of ME/CFS have revealed irregularities in energy, amino acid, nucleotide and nitrogen metabolism^7^, as well as inconsistent disturbances in neurotransmitter-related pathways and lipid metabolism. In a mass spectrometric study of 45 ME/CFS subjects and 39 healthy controls, Naviaux *et al*. reported abnormalities in levels of phospho- and sphingolipids, cholesterol, branched-chain amino acids, vitamins, proline/glutamate and mitochondrial metabolites^8^. In a study of 67 ME/CFS patients and 66 healthy controls, Yamano and colleagues assayed 144 metabolites and reported abnormalities in levels of metabolites related to glycolysis, the tricarboxylic acid cycle and the urea cycle, but not in glutamine metabolism^9^. Fluge *et al*. reported evidence of perturbations consistent with impaired pyruvate dehydrogenase function linked to TCA cycle-based impairments in energy production^10^. Nuclear magnetic resonance (NMR) spectroscopy has also revealed metabolomic abnormalities consistent with altered gluconeogenesis, potential inhibition of glycolysis and impaired oxidative stress response^11^. Another NMR study found that, as compared with controls, glutamine and ornithine serum levels in ME/CFS were lower, and correlated with metabolites linked to the urea cycle^12^. A pilot study focused on plasma from women with ME/CFS revealed alterations in energy-related metabolic compounds and pathways^13^. We have reported fecal microbiome analyses and proposed a model wherein intestinal dysbiosis may contribute to bacterial metabolic disturbances that are distinct between ME/CFS subgroups defined by the presence or absence of IBS^14^.

Here we report targeted and untargeted analyses of 562 molecules representing primary metabolites, biogenic amines, lipid complexes and oxylipins in plasma of ME/CFS patients and controls. We also describe linkage of the resulting metabolomic data to a fecal metagenomic dataset and clinical data. As in our previously reported metagenomic analyses, we found that metabolomic profiles differ not only between ME/CFS patients and controls but also between ME/CFS patients who do or do not have IBS.

## Results

### Study population characteristics

Subjects included 50 ME/CFS cases who met the criteria^15,16^ for ME/CFS and 50 matched healthy controls recruited at four sites across the United States (New York, NY; Salt Lake City, UT; Incline Village, NV; and Miami, FL). Subject demographics are shown in Table 1. The same subjects were enrolled as in our previous study^14^. Cases included 41 female and 9 male ME/CFS patients (mean age 51.1 years; standard error of the mean [SEM] 1.6). Controls included 41 female and 9 male subjects (mean age 51.3 years; SEM 1.6). All case and control samples were collected between June 22, 2014 and October 27, 2014. Irritable bowel syndrome (IBS) was diagnosed in 24 of the 50 ME/CFS patients (48%). One of the 50 control subjects reported a diagnosis of IBS (2%).

**Table 1:** Characteristics of the study cohort.

Demographics		ME/CFS (n = 50)	Controls (n = 50)
Sex	Female	41	41
	Male	9	9
Age	Mean (±SEM)	51.081 (±1.607)	51.320 (±1.620)
	Median (range)	53.607	52.93
		(20.493–66.500)	(21.040–67.869)
Race	White	49	48
	Asian	1	1
	Other	0	1
Ethnicity	Not Hispanic or Latino	46	45
	Hispanic or Latino	4	5
Site of Collection	New York, NY	14	14
	Salt Lake City, UT	14	15
	Sierra, NV	12	12
	Miami, FL	10	9
Season of Collection	Summer	27	26
	Fall	23	24
IBS Co-morbidity	with IBS	24	1
	without IBS	26	49
BMI	High BMI (>25 kg/m^2^)	28	22
	Normal BMI (<25 kg/m^2^)	22	28
Duration of ME/CFS	Long duration (>3 years)	46	N/A
	Short duration (<3 years)	4	N/A

ME/CFS: myalgic encephalomyelitis/chronic fatigue syndrome, IBS: irritable bowel syndrome, BMI: body mass index, SEM: standard error of the mean, N/A: not applicable.

### Metabolomic dataset

**Figure 1:**
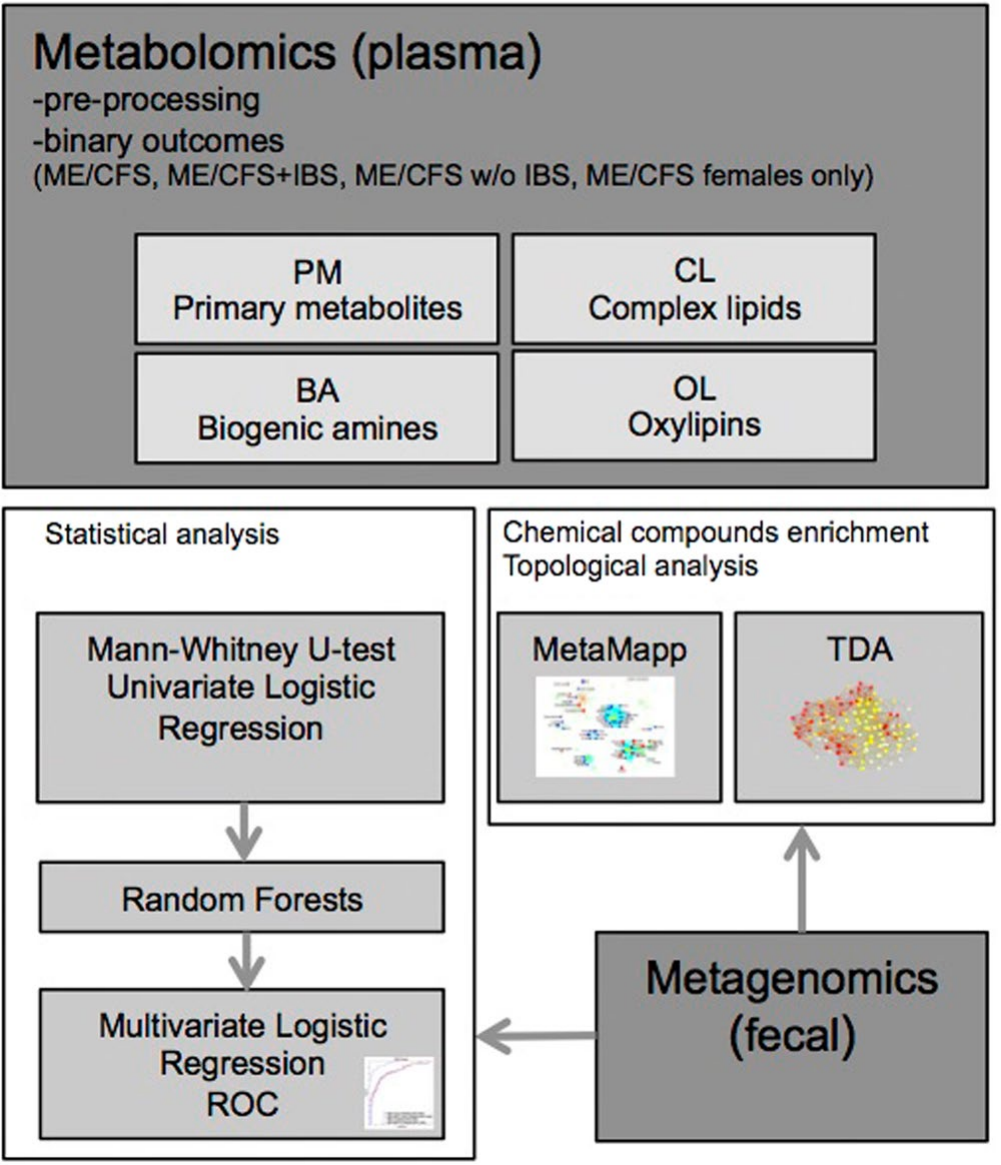
Schematic figure describing the metabolomic and metagenomic analysis pipeline. Metabolomic data was pre-processed and compared between ME/CFS patients vs. controls, ME/CFS with IBS (ME/CFS + IBS) vs. controls and ME/CFS without IBS vs. controls, and female group only. Targeted and untargeted mass spectrometry platforms yielded data for 111 primary metabolites (PM), 103 biogenic amines (BA), 302 complex lipids (CL) and 46 bioactive oxylipins (OL). Statistical analyses were performed with Mann-Whitney U test and adjusted univariate logistic regression modeling on all metabolites except for exposomes/vitamins. After removal of 5-methoxytryptamine, metabolites with a p-value below 0.05 in both U test and univariate logistic regression were ranked with random forests. The top 10 random forests-ranked metabolites were used as predictors in the predictive multivariate logistic regression models. The goodness-of-fit and predictive performance for the predictive models were measured with ROC curves. Biochemical set enrichment and topological analyses were performed with MetaMapp and TDA software, respectively. Metagenomic data were incorporated to better understand the correlation between metabolites and bacterial abundance profiles.

Targeted and untargeted mass spectrometry platforms yielded data for 562 metabolites comprising 111 primary metabolites (PM), including those related to tryptophan metabolism, sugars, hydroxyl acids, ketone bodies and other energy-metabolism compounds; 103 biogenic amines (BA) including branched and unbranched acylcarnitines, trimethylamine N-oxidase (TMAO), choline and amino acids; 302 complex lipids (CL) including mono- and diacylglycerides, fatty acids, ceramides, sphingomyelins and phospholipids; and 46 bioactive oxylipins (OL), steroids and bile acids. Figure 1 shows the pipeline for metabolomic data processing and the statistical methods used.

### ME/CFS is associated with an altered metabolomic profile

Exposome and vitamin metabolites (exogenous environmental compounds, Table S1) were excluded from our biomarker analysis because vitamin supplements, medications and diet may impact metabolite levels independent of disease status. We used the nonparametric Mann-Whitney U test (p < 0.05) and adjusted univariate logistic regression model (p < 0.05) to select plasma metabolites potentially altered in ME/CFS amongst the remaining 514 metabolites. The logistic regression model was adjusted for frequency matching variables, body mass index (BMI) and IBS. From this subset of metabolites, we selected the top 10 potential ME/CFS biomarkers by random forests method^17^. 5-methoxytryptamine (5-MT) was excluded from random forests analysis as it is confounded by the use of antidepressants in 50% of cases (Table S2). Potential biomarkers were used to test for accuracy of ME/CFS prediction in a multivariate logistic regression model.

Among the top plasma biomarkers differentiating ME/CFS patients from controls were decreased levels of betaine, complex lipids (lysophosphatidylcholine [LPC], phosphatidylcholine [PC]) and sphingomyelin (SM), and increased levels of triglycerides (TG), α-N-phenylacetyl-glutamine, ε-caprolactam and urobilin (Table S2). Set enrichment analysis of the results of logistic regression models revealed that ME/CFS subjects had reduced levels of PCs and dysregulation of the choline-carnitine pathway (Table 2).

**Table 2:** Chemical enrichment analysis.

Enrichment set name	Set size	Direction in ME/CFS	ME/CFS vs. controls			ME/CFS + IBS vs. controls			ME/CFS w/o IBS vs. controls		
			compound level p < 0.05			compound level p < 0.05			compound level p < 0.05		
			# compounds	enriched *p*	adjusted *p*	# compounds	enriched *p*	adjusted *p*	# compounds	enriched *p*	adjusted *p*
**carnitine-choline**	**3**	**Decreased**	**3**	**0.002**	**0.017**	**3**	**0.001**	**0.013**	**3**	**0.001**	**0.016**
**PC**	**115**	**Decreased**	**24**	**0.001**	**0.017**	9	0.914	1.000	**25**	**<0.001**	**0.006**
**TG**	**60**	**Increased**	11	0.084	0.580	**16**	**<0.001**	**0.004**	10	0.138	0.725
**ceramide**	**25**	**Increased**	3	0.591	1.000	**8**	**0.003**	**0.021**	3	0.569	1.000
**PE**	**18**	**Increased**	2	0.655	1.000	**5**	**0.036**	0.189	2	0.637	1.000
amino acid	61		4	0.952	1.000	1	0.999	1.000	3	0.983	1.000
oxylipin	47		2	0.984	1.000	0	1.000	1.000	1	0.998	1.000
exposome	45		1	0.997	1.000	3	0.890	1.000	1	0.997	1.000
fatty acid	31		2	0.905	1.000	0	1.000	1.000	1	0.980	1.000
SM	30		2	0.894	1.000	2	0.859	1.000	3	0.698	1.000
energy	27		2	0.856	1.000	1	0.958	1.000	2	0.843	1.000
LPC	25		3	0.591	1.000	4	0.283	0.742	3	0.569	1.000
carnitine	19		2	0.685	1.000	0	1.000	1.000	2	0.668	1.000
sugar	11		0	1.000	1.000	0	1.000	1.000	0	1.000	1.000
sugar alcohol	9		0	1.000	1.000	1	0.647	1.000	0	1.000	1.000
cholesterol	9		2	0.292	1.000	2	0.254	0.742	2	0.280	1.000
neutral lipid	8		1	0.640	1.000	1	0.604	1.000	1	0.628	1.000
one-carbon/nicotinate	6		0	1.000	1.000	2	0.131	0.457	0	1.000	1.000
neurotransmitter	5		2	0.110	0.580	2	0.093	0.393	2	0.105	0.725
nucleotide	4		0	1.000	1.000	0	1.000	1.000	0	1.000	1.000
vitamin	4		1	0.399	1.000	1	0.369	0.862	1	0.389	1.000

Cells in bold represent significant chemical enrichment in ME/CFS vs. control, ME/CFS + IBS vs. control and ME/CFS without IBS vs. control^20^. ME/CFS: myalgic encephalomyelitis/chronic fatigue syndrome, IBS: irritable bowel syndrome, LPC: lysophosphatidylcholine, TG: triglyceride, PC: phosphatidylcholine, SM: sphingomyelin, PE: phosphatidylethanolamin.

**Figure 2:**
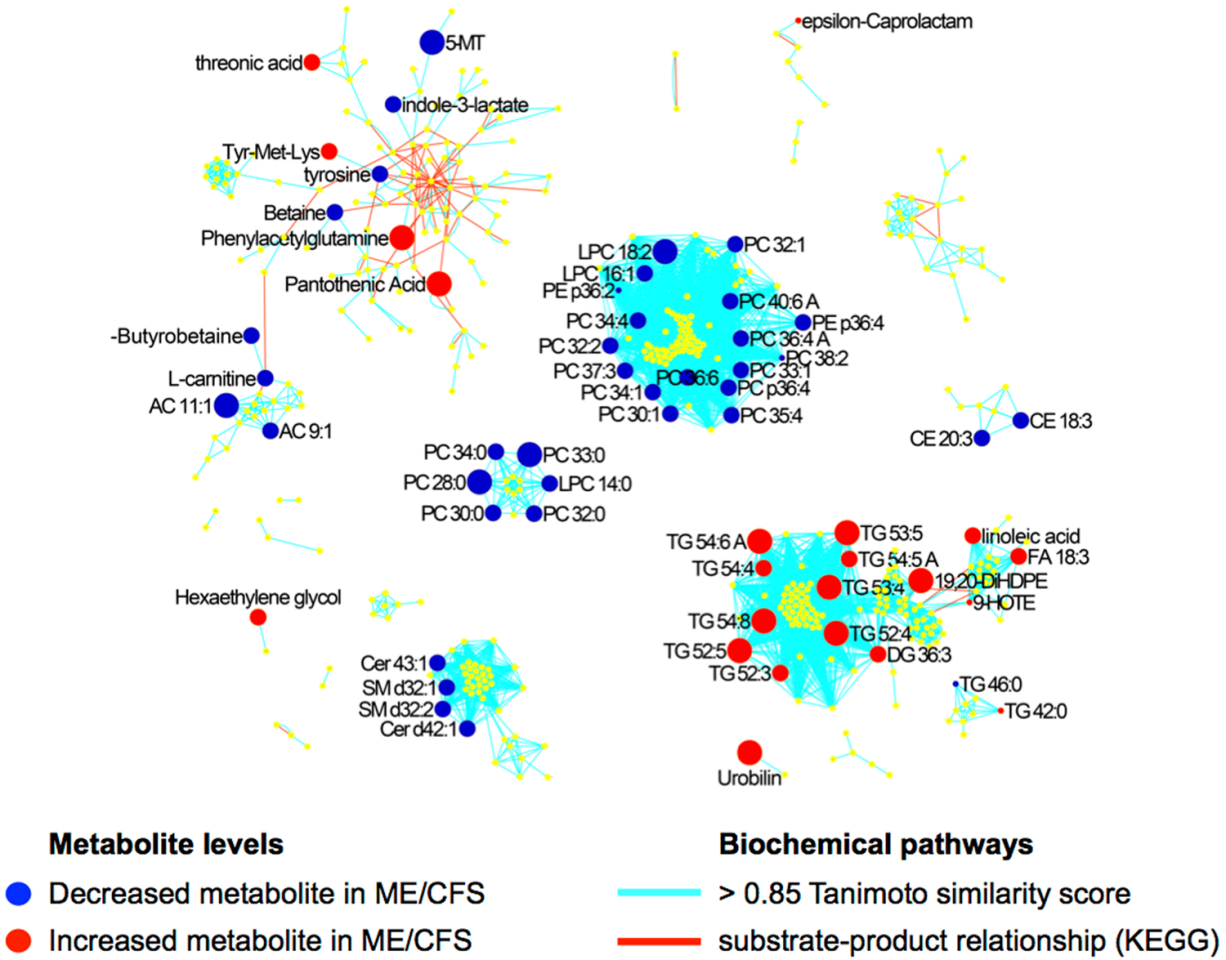
Metabolites differentiate ME/CFS and controls. A MetaMapp network of all identified metabolites in the ME/CFS cohort was constructed by Tanimoto chemical similarity and Kyoto Encyclopedia of Genes and Genomes (KEGG) reaction pairs. Each node represents a metabolite. Up-regulated nodes are marked red and down-regulated nodes are marked blue. Node size reflects the magnitude of the effect. Only the compounds that pass a p-value cutoff of 0.05 are labeled. Red lines show the biochemical reactions and blue lines are chemical similarity scores above 0.85 Tanimoto similarity coefficients. The network was created using www.metamapp.fiehnlab.ucdavis.edu^18^ and visualized in Cytoscape using the organic layout algorithm. MetaMapp identified perturbations in tryptophan metabolism, carnitine shuttle/energy homeostasis and complex lipids. Metabolites representing the tryptophan and carnitine pathway were decreased in ME/CFS compared to controls. In contrast, threonic acid, amino acids (tyrosine, methionine and lysine), phenylacetylglutamine, pantothenic acid, hexaethylene glycol and ε-caprolactam were enriched in ME/CFS. Lipid analyses showed that whereas metabolites representing the SM, Cer/CE and PC/LPC pathways were decreased in ME/CFS, TG pathways were enriched. *5-MT: 5-methoxytryptamine, 9-HOTE: C18:3n3, 19,20-DiHDPE: C22H34O4, AC: acylcarnitine, Cer/CE: ceramide, DG: diacylglycerol, FA: fatty acid, LPC: lysophosphatidylcholine, PC: phosphatidylcholine, PE: phosphatidylethanolamine, SM: sphingomyelin, TG: triglyceride, Tyr Met Lys: tyrosine methionine lysine*.

Results from logistic regression analysis were supported by a MetaMapp analysis of all metabolites including vitamins and neurotransmitters. MetaMapp software maps metabolites by their biochemical and chemical relationships into a metabolic network graph^18^. The MetaMapp in Fig. 2 describes increased TGs and fatty acids and decreased carnitines, ceramides, SMs and phosphatidylcholines in ME/CFS cases compared to controls. The MetaMapp also demonstrated decreased indole moiety-containing metabolites such as 5-MT, tyrosine and indole-3-lactate and increased vitamin B5 (pantothenic acid) in ME/CFS subjects.

### IBS co-morbidity is associated with altered metabolic profiles in ME/CFS patients

In previous work with these same subjects, IBS co-morbidity was the strongest factor driving separation in topological networks based on fecal metagenomic data (bacterial relative abundance and predicted bacterial metabolic pathways)^14^. Chemical enrichment analysis of plasma metabolites revealed that metabolomic profiles of ME/CFS patients with IBS were distinguished from controls by levels of TG, ceramides, phosphatidylethanolmines (PE) and metabolites in the carnitine-choline pathway (Table 2, Fig. S1A).

ME/CFS patients without IBS co-morbidity showed disturbances in PCs and carnitine-choline pathways, similar to the disturbances found in the overall ME/CFS cohort (Table 2, Fig. S1B). Analysis of plasma metabolites by Mann-Whitney U test, logistic regression and random forests identified increased levels of ceramides, TGs, PE, 5-methylthioadenosine, mannitol and betaine as well as decreased levels of LPCs and γ-butyrobetaine in ME/CFS patients with IBS versus controls (Table S3A). In ME/CFS patients without IBS, we found decreased levels of tyrosine and PCs and increased levels of TGs and Tyr-Met-Lys compared with controls (Table S3B). Relative to controls, all ME/CFS groups (all ME/CFS cases, ME/CFS with IBS and ME/CFS without IBS) had decreased levels of carnitine-choline metabolites (Table 2). ME/CFS patients with IBS have a distinct metabolomic profile (Fig. S2A, Table S4A).

### ME/CFS is associated with an altered metabolomic profile in women

The metabolic profiles in women with ME/CFS were similar to those seen in all ME/CFS cases (Fig. S2B, Table S4). The levels of complex lipids (LPC and PC) were decreased and the levels of TGs were increased in ME/CFS vs. controls (Table S3C). Among the top 10 plasma biomarkers differentiating the female ME/CFS patient group from the female control group were decreased levels of indole-3-lactate and γ-butyrobetaine. Indole-3-lactate and γ-butyrobetaine were also reduced in the all ME/CFS group but were not amongst the top 10 plasma biomarkers.

### Selected metabolites as a potential diagnostic tool in ME/CFS

#### ME/CFS cases vs. controls

We used binary logistic regression to test the sensitivity and specificity of the top 10 ME/CFS biomarkers that were identified through random forests analyses (Table S2). These metabolites distinguished ME/CFS subjects from controls with a high degree of accuracy (receiver operating characteristic (ROC) and area under the curve (AUC) = 0.960, cross-validated AUC = 0.820) (MET, Fig. S3A). Eight bacterial species were found to predict ME/CFS in random forests analyses in our previous study^14^ (*Coprococcus (C.) catus, Pseudoflavonifractor (P.) capillosus, Dorea (D.) formicigenerans, Faecalibacterium (F.) prausnitzii*, *Clostridium (C.) asparigiforme, Sutterella (S.) wadsworthensis, Alistipes (A.) putredinis* and *Anaerotruncus (A.) colihominis*), with a cross-validated AUC value of 0.745. A model that integrates metagenomic and metabolomic data provided better predictive performance (MET + BACT: ROC AUC = 1.000, cross-validated AUC = 0.836; Fig. S3A) than either metagenomic or metabolomic data alone. We tested for selection bias using Lasso (least absolute shrinkage and selection operator) with cross-validation on all metabolites and achieved a similar AUC (MET cross-validated AUC = 0.820 vs. MET Lasso AUC = 0.822).

#### IBS subgroups vs. controls

Using metabolites selected by the random forests method, a binary multivariate logistic regression method was used to predict ME/CFS subgroups from controls based on IBS co-morbidity. In ME/CFS and IBS (Table S3A, Fig. S3B), the top 10 metabolites distinguished ME/CFS with IBS from control subjects with a high degree of accuracy (ROC AUC = 0.877, cross-validated AUC = 0.754). Nine bacterial species were selected to predict ME/CFS + IBS in random forests analyses in our previous study (*F. cf., F. prausnitzii, Bacteroides (B.) vulgatus, A. putredinis, C. catus, Anaerostipes (A.) caccae, D. formicigenerans, A. colihominis* and *C. asparagiforme*), with a cross-validated AUC value of 0.791. A model that integrates metagenomic and metabolomic data provided better predictive performance (MET + BACT: ROC AUC = 1.000, cross-validated AUC = 0.824; Fig. S3B) than either metagenomic or metabolomic data alone.

In ME/CFS without IBS (Table S3B, Fig. S3C), the top 10 metabolites also distinguished ME/CFS without IBS from control subjects with a high degree of accuracy (ROC AUC = 0.975, cross-validated AUC = 0.839). Seven bacterial species were selected to predict ME/CFS + IBS in random forests analyses in our previous study (*B. caccae, P. capillosus, Parabacteroides (P.) distasonis, B. fragilis, Prevotella (P.) buccalis, B. xylanisolvens* and *D. formicigenerans*) and they yielded a cross-validated AUC value of 0.754. A model that integrates metagenomic and metabolomic data provided better predictive performance (MET + BACT: ROC AUC = 1.000, cross-validated AUC = 0.880; Fig. S3C) than either metagenomic or metabolomic data alone.

### Plasma metabolomic profiles, fecal bacterial abundance profiles, and symptom severity scores in ME/CFS

The relative abundance of bacterial species in subjects’ feces varied with their plasma metabolomic profiles. Decreased betaine was associated with decreased *A. colihominis*. Decreased PC 30:0 correlated with decreased *A. putredinis* (Table S4), a bacterium known to produce sulfonolipids - unusual sphingolipids structurally related to ceramides^19^. Sphingolipids are known to maintain bacterial survival and promote stress resistance^20^.

In ME/CFS with IBS, 5-methylthioadenosine (a metabolite derived from S-adenosylmethionine as a by-product of polyamine biosynthesis that can be toxic to mammalian cells^21^) was associated with functional impairment. Ceramides were associated with increased physical fatigue (Table S4). Decreased γ-butyrobetaine correlated with increased *Faecalibacterium*, a bacterium known to play an important role in the production of colonic butyrate, a by-product of fermentation with known beneficial impacts on intestinal barrier function and anti-inflammatory effects^22,23^.

In ME/CFS without IBS, decreased tyrosine correlated with decreased *P. distasonis*. Increased TG 54:6 A was correlated with *D. formicigenerans* and TG 54:8 was correlated with decreased *B. caccae* and *D. formicigenerans*. *Parabacteroides* and *Bacteroides* species have the ability to convert complex polysaccharides into energy sources^24^. *D. formicigenerans* is a carbohydrate-fermenting bacteria producing formic acid and lactate^25^.

### TDA analysis for network identification

**Figure 3:**
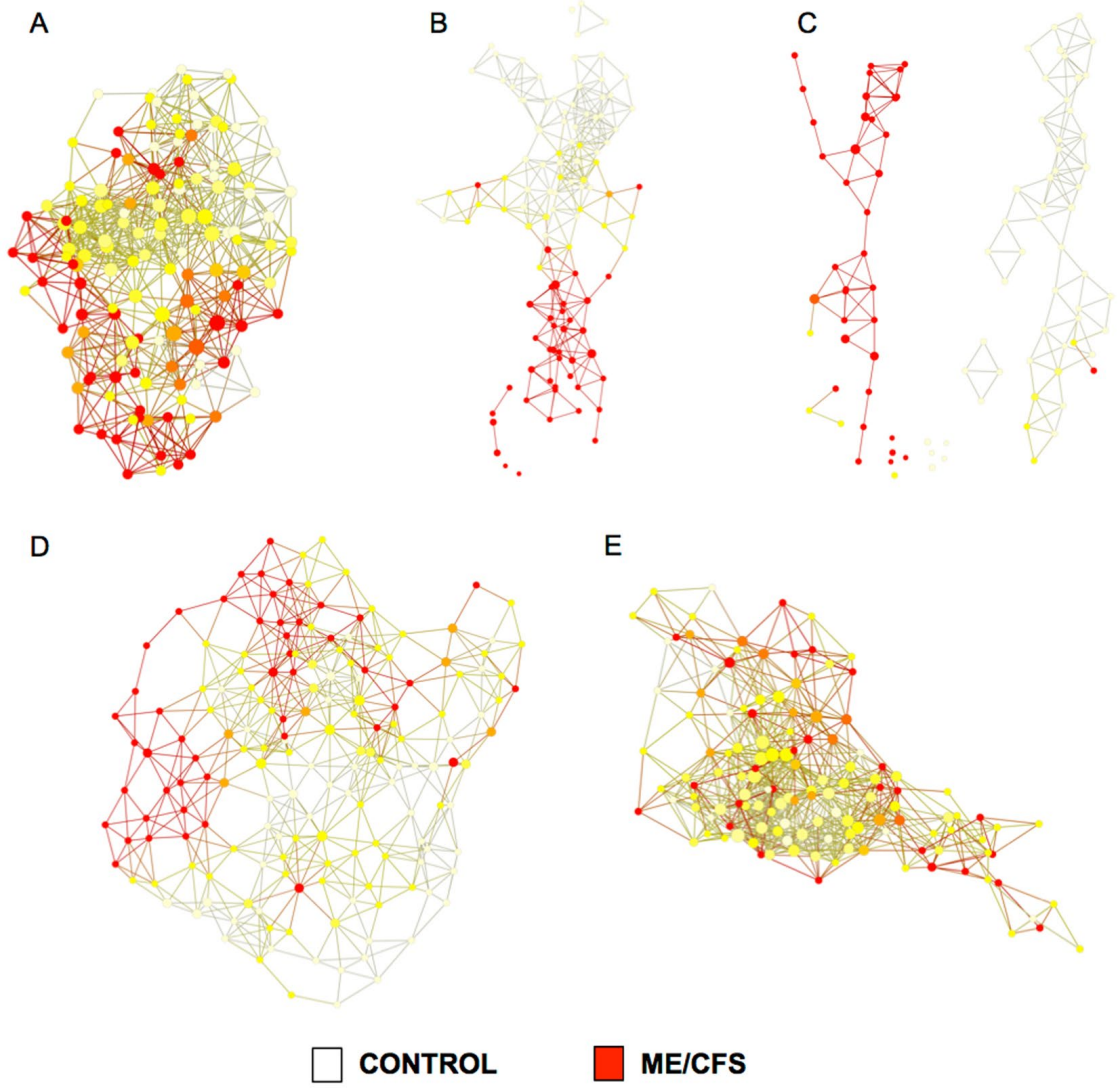
Topological data analysis (TDA) revealed altered metabolomic and metagenomic profiles in ME/CFS. The color scheme represents the strength of association with ME/CFS diagnosis (white: strongly associated with control, red: strongly associated with ME/CFS). Each node in a network comprises 1 or more subject(s) who share variables in multiple dimensions. Lines connect network nodes that contain shared variables and subjects. Unlike traditional network models wherein each node reflects only a single sample, the size of a node in the topological network is proportional to the number of variables with a similar profile. (**A**), (**B**) and (**C**) integrate plasma metabolomic, fecal metagenomic and plasma immune profiles, and symptom severity scores using the Jaccard metric to define multidimensional subgroups. Irrespective of the lenses used [(**A**) neighborhood lenses NL1 and NL2, (**B**) MDS coordinate 1 and 2, and (**C**) metric PCA 1 and 2], ME/CFS and control samples formed distinct networks. ME/CFS and control samples also formed distinct networks in TDA based on either fecal bacterial relative abundance or plasma metabolomic data in isolation using a variance normalized Euclidean distance metric with neighborhood lenses (NL1 and NL2). Fecal bacterial relative abundance features (**D**) were stronger drivers of the network distinction than plasma metabolomic features (**E**).

Topological data analysis (TDA) based on plasma metabolomic and fecal metagenomic data and clinical symptom severity identified networks that distinguished ME/CFS cases from controls (Fig. 3A–C). Profiles of bacterial relative abundance were stronger drivers of network distinction than plasma metabolites (Fig. 3D,E).

## Discussion

We recently reported that ME/CFS patients with IBS, ME/CFS patients without IBS and normal control subjects have group-specific differences in fecal microflora^11^. We have extended characterization of these same subjects through targeted and untargeted metabolomic analyses of plasma and integrated analysis of plasma metabolomic, fecal metagenomic and clinical data.

The Naviaux group previously reported decreased plasma levels of SM, ceramides and PLs with the exception of PC 18:1 and PC 22:6, which were increased^8^. Tomic *et al*. reported increased plasma levels of TGs^26^. Germain *et al*. reported metabolomic biomarkers of disturbed amino acid, energy, sugar and fatty acid metabolism in ME/CFS patients^13^. Neither group examined the impact of IBS on metabolomic profiles in ME/CFS. Our analysis confirmed decreased levels of phospholipids and SM, and increased levels of TGs, but differed with respect to specific compounds. We did not find case-control differences in levels of PC 18:1 and PC 22:6. Nor did we find a consistent decrease in ceramide levels. Whereas ME/CFS without IBS had decreased levels of ceramides d43:1 and d42:1, ME/CFS with IBS had increased levels of six ceramide species: d36:1, d40:0, d42:0, d 34:1, d38:1 and d40:1.

Ceramide is a waxy lipid implicated in suppression of electron transport, insulin and leptin resistance and apoptosis^27,28^. Previous studies reported that ceramides might be involved in the pathology of IBS and metabolic disorders^29,30^. Increased levels of lipopolysaccharides (LPS) associated with an altered gut microbiome^31^ may trigger the activation of sphingomyelinases (SMAse) and the hydrolysis of SM to produce ceramides. Ceramides are toxic to many cell subtypes via the production of reactive oxygen species and may play a role in gut barrier dysfunction and increased gut permeability. Increased levels of ceramides were reported in mucosal samples from IBS patients^29^ as well as in plasma and tissue samples in diabetes, cardiomyopathy, insulin resistance, atherosclerosis and steatohepatitis. Blocking SMAse to decrease ceramide levels may be therapeutic in reducing inflammation^32^. Patients with ME/CFS and IBS also had higher plasma mannitol levels. We speculate that mannitol may increase permeability of both the gut mucosa and the blood-brain barrier resulting in trafficking of molecules such as cytokines and neurotransmitters that contribute to disease.

Similar to Armstrong and colleagues^11^, we found alterations in metabolites associated with mitochondrial energy metabolism. Previous study reported significant decreases in TCA cycle metabolites related to energy metabolism in ME/CFS patients^9^. The reported dysfunction impacted carnitine metabolism and ATP/energy metabolism in the muscle of ME/CFS patients^11^. Carnitine is an important supplement that transfers acyl-CoA group into the mitochondrial matrix and participates in fatty acid β-oxidation (TCA cycle, ATP production and energy metabolism). Studies of carnitine levels in serum and plasma in ME/CFS have been inconclusive, with some groups reporting reductions whereas others find normal levels^33,34^. In an open label study of 30 patients, acetyl-carnitine supplements were reported to improve fatigue and cognitive function in up to 59% of patients with ME/CFS^35^. In our study (Table 2), compounds in the choline-carnitine pathway were decreased in ME/CFS patients regardless of their IBS status.

Our results are consistent with earlier reports that suggest that metabolites linked to lipid and energy metabolism are affected in ME/CFS. They extend earlier work by demonstrating that ME/CFS subjects with IBS co-morbidity have a distinct metabolomic profile compared to subjects without IBS and controls.

Many ME/CFS patients in our cohort take vitamin B supplements (26/50, 52%) that have the potential to increase levels of pantothenic acid. Use of vitamin B supplements was associated with higher levels of pantothenic acid and lower fatigue scores (data not shown). However, the numbers of samples were not sufficient to test for a significant relationship. Plasma levels of 5-MT, a compound related to tryptophan, serotonin and melatonin metabolism, were decreased in ME/CFS; however, this finding was confounded by the use of selective serotonin reuptake inhibitors (SSRIs) or other antidepressants (serotonin-norepinephrine reuptake inhibitors [SNRIs] and tricyclic antidepressant [TCAs]) in 25 of 50 (50%) ME/CFS subjects vs. 6 of 50 (12%) healthy controls. Prior metabolomic studies have shown reduced levels of plasma 5-MT with chronic, but not acute, SSRI administration^36,37^. In human cells, 5-MT is an important metabolite involved in two-step conversion pathways between serotonin (5-hydroxytryptamine) and melatonin (N-acetyl-5-methoxytryptamine)^38,39^. Reduced levels of serotonin transporters, which play a role in regulating serotonin levels at synapses, have previously been reported in ME/CFS^40^.

Correlation studies suggested potential relationships between the 5-MT neurotransmitter metabolites and ME/CFS severity symptoms including impaired cognitive function, sleeping disturbances and overall elevated ME/CFS fatigue scores. Additional studies with larger subject numbers will be required to address whether plasma levels of pantothenic acid or 5-MT can be correlated with symptoms and to determine whether there is a subset of ME/CFS patients who might be predicted to benefit from drugs that modulate the associated pathways.

Women accounted for 41 of 50 ME/CFS subjects (82%) and 41 of 50 (82%) control subjects in our study. Given that the vast majority of cases and controls were women, it is not surprising that findings in women with ME/CFS were similar to findings in the overall ME/CFS case group. Both had lower levels of complex lipids (LPC and PC) and higher levels of TGs than controls. The cohort contains only 9 ME/CFS men and 9 control men; thus, we do not have the power needed to address differences between males and females. This difference in prevalence by sex in our cohort is characteristic of ME/CFS (typical 4:1 ratio of women to men).

Our predictive modeling distinguished ME/CFS patients from controls with high accuracy; however, it needs to be verified in another independent study.

ME/CFS is a heterogeneous disorder. Identification of ME/CFS subgroups characterized by specific metabolomic profiles that integrate primary metabolites, biogenic amines, complex lipidomics and oxylipins may enable delineation of subtypes and lead to specific diagnostic and therapeutic strategies.

## Methods

### Study design

We report association modeling, biomarker discovery, biochemical enrichment analysis and topological network visualization of plasma metabolomic, fecal bacterial metagenomic and clinical data from 50 ME/CFS patients and 50 healthy controls. 562 plasma metabolites were assessed using targeted and untargeted mass spectrometry platforms. Figure 1 shows the pipeline for metabolomic data processing and the statistical methods used.

### Study population and plasma collection

Subjects included 50 cases and 50 controls from the Chronic Fatigue Initiative (CFI) cohort^41^ recruited at four sites across the US who met the 1994 CDC Fukuda^15^ and/or Canadian consensus criteria for ME/CFS^16^. Controls were frequency-matched to cases on age, sex, race/ethnicity, geographic/clinical site and season of sampling^42^. All ME/CFS subjects (n = 50) completed standardized screening and assessment instruments including medical history and symptom rating scales, had a physical examination and provided blood samples. Controls (n = 50) of the CFI cohort study^42^ had been found to be free of: self-reported ME/CFS or ME/CFS symptoms or other conditions deemed by the recruiting physician to be non-representative of a healthy control population including substance abuse in the prior year and any history of self-reported psychiatric illness; antibiotics in the prior three months; immunomodulatory medications in the prior year; and clinically significant findings on physical exam or screening laboratory tests. All participants provided informed written consent in accordance with protocols approved by the Institutional Review Board at Columbia University Medical Center. All participants consented to phlebotomy, collection of stool samples and completion of clinical questionnaires.

Blood samples were collected into BD VacutainerTM Cell Preparation Tubes (CPT with sodium citrate anticoagulant) between June and October 2014, and centrifuged to pellet red blood cells. The plasma was shipped to Columbia University at 4 °C. After aliquoting, samples were stored at −80 °C until thawed for metabolomic analyses. All samples were analyzed within 2 years of collection.

### Clinical assessments and medical history

Clinical symptoms and baseline health status were assessed on the day of physical examination and biological sample collection from both cases and control subjects using the following surveys: the Short Form 36 Health Survey (SF-36), the Multidimensional Fatigue Inventory (MFI), DePaul Symptom Questionnaire (DSQ) and Pittsburgh Sleep Quality Index (PSQI^43^). Table S5 describes the individual surveys with the specific questions listed. The SF-36 includes the following subject-reported evaluations about current health status: physical and social functioning, physical and emotional limitations, vitality, pain, general health perceptions and mental health change^44^. The MFI comprises a 20-item self-reported questionnaire focused on general, physical and mental fatigue, activity and motivation^45^. Cognitive function was tested based on the DSQ questionnaire data^46^ and was scored using a standard cognitive disturbance definition as well as a modified definition based on a subset of questionnaire variables. Sleeping disturbances linked to ME/CFS were tested and scored based on DSQ and PSQI questionnaire items. Each instrument was transformed into a 0–100 scale to facilitate combination and comparison, wherein a score of 100 is equivalent to maximum disability or severity and a score of zero is equivalent to no disability or disturbance.

IBS co-morbidity was based on self-reported diagnosis of IBS on the medical history form. IBS was diagnosed in 24 of the 50 ME/CFS patients (48%). One control subject of out 50 reported a diagnosis of IBS (2%). Three ME/CFS cases and 1 control were newly diagnosed with IBS.

### Metabolomics

Three untargeted metabolomic assays and 1 targeted assay for 562 metabolites from over 20 biochemical pathways were performed with gas chromatography time-of-flight (GCTOF) and liquid chromatography–tandem mass spectrometry (LC-MS/MS) instruments by the West Coast Metabolomics Center at University of California, Davis, USA. Sample preparation and metabolomic analyses were described previously^47–49^. Mass spectrometry metabolomic data have been deposited in the Metabolomics Workbench (http://www.metabolomicsworkbench.org) with project identifier PR000576.

### Statistical analyses

Mass spectrometry data were initially filtered for internal standard metabolites (quality control spike-in metabolites) and unannotated/unknown metabolites. Annotated metabolites were further filtered out if more than 50% of samples (50 individuals out of 100) showed non-detectable or missing profiles. For each metabolite, we created a dummy variable for whether the values were missing, and ran chi-squared tests between this dummy variable and the ME/CFS status variable. We found no significant difference in prevalence of missing metabolite values between ME/CFS and controls. For each of the remaining metabolites, missing values were imputed using 50% of its smallest available value. Normalization was performed by dividing each metabolite profile by the total sum of metabolite intensities per sample^50^. Base-10 log transformation was applied to limit outlier effect. Keeping features on a positive domain, all data points were multiplied by a factor of 1.0e + 09 before log transformation. Data for each metabolite were then scaled by the control standard deviation.

The nonparametric Mann-Whitney U test and adjusted univariate logistic regression were applied to identify potential predictors differentiating ME/CFS from controls. For the binary outcome of IBS subgroups vs. controls, logistic regression models were adjusted for all frequency matched variables and BMI. For the binary outcome of ME/CFS vs. controls, additional adjustment for IBS was included. The metabolites with a p-value below 0.05 in both U test and adjusted univariate logistic regression were then used to develop a representative set of variables by random forests^17^. The top 10 random forests ranked metabolites were fitted as predictors in the predictive multivariate logistic regression models^51,52^. Table S6 shows the descriptive value of chemical compounds differed in ME/CFS, subgroups and ME/CFS female subjects only. 5-MT was excluded from random forests analysis as it is confounded by the use of antidepressants in 50% of cases. In-sample receiver operating characteristic (ROC) curves were plotted and area under the curve (AUC) was measured to assess the goodness-of-fit of the models. To examine the predictive performance of the multivariate logistic regression models, random resampling cross-validation was performed with 1000 iterations. Data were randomly split into a training set (80%) and a test set (20%) within each iteration.

Correlations between bacteria, metabolites and disease scores were examined using Spearman correlations.

Data were analyzed and visualized with Matlab (R2013a, The Mathworks Inc., MA), R (version 3.3.3) and SPSS (version 24, IBM, NY). All p-values were 2-tailed.

### MetaMapp network mapping and enrichment analysis

MetaMapp networks were created using the method described in the ref.^18^. Cytoscape software version 3.4.0 was used to visualize the networks with overlaid statistical results. Networks were visualized using the organic layout algorithm. Node size was mapped to fold-change differences in the case vs. control groups. Node color was mapped to the direction of the between-group differences. Structurally identified metabolites were grouped into 21 chemical groups. Metabolites were manually classified because many compounds, including complex lipids, are poorly covered in pathway maps from major databases such as KEGG, Reactome or MetCyc. Metabolite groups were used as input for an enrichment analysis using Fisher exact test in R. P-values for the enrichment were corrected for multiple hypothesis testing using the Benjamini-Hochberg false discovery rate (FDR) method^53^ controlling the FDR at 0.05 level.

### Topological data analyses

Metagenomic and metabolomic data including plasma metabolite levels, bacterial composition and inferred metabolic pathways, plasma immune profiles and health symptom severity scores were integrated for TDA using the AYASDI platform (Ayasdi, Menlo Park, California). AYASDI represents high-dimensional, complex biological data sets as a structured 3-dimensional network^54^. Each node in the network comprises 1 or more subject(s) who share variables in multiple dimensions. Lines connect network nodes that contain shared data points. Unlike traditional network models wherein each node reflects only a single sample, the size of a node in the topological network was proportional to the number of variables with a similar profile. We built a network comprised of 100 samples and 1900 variables (562 variables representing plasma metabolites, 574 representing fecal bacterial relative abundances at different taxonomic levels, 586 variables representing predicted bacterial metabolic pathways, 61 variables reflecting immune molecules, 81 variables representing different ME/CFS fatigue and other symptom score/health questionnaire items and information on co-morbidities; and 36 demographic variables). All variables were weighted equally. Jaccard and variance-normalized Euclidean distance methods were used as the distance metrics; a range of filter lenses (neighborhood lens 1 and 2, MDS coordinate 1 and 2, Metric PCA 1 and 2) was used to identify networks. A metric represents a notion of similarity (or distance) between rows in the data. The Jaccard metric measures the dissimilarity of asymmetric information on non-binary variables. Each row must be a collection of objects (no order necessary) and this metric computes the Jaccard score (1 - intersection/union) between the two sets. The Jaccard metric treats the columns as delimiters. The rows are treated as a list of objects in the set. The variance-normalized Euclidean metric is a variant on the Euclidean that accounts for the scale choices. For each variable, this metric finds its mean and standard deviation, and rescales the value of the coordinate by subtracting the coordinate mean and dividing by the corresponding standard deviation. A lens is a filter that converts the dataset into a vector. The neighborhood lenses generate an embedding of high-dimensional data into two dimensions by embedding a k-nearest neighbors graph of the data. A k-nearest neighbors graph is generated by connecting each point to its nearest neighbors. The MDS coordinate 1/2 and Metric PCA 1/2 lenses compute a variant of PCA coordinate lenses for data that does not use the Euclidean metric. The AYASDI maps the data into a Euclidean space using the rows of the distance matrix as the coordinates and then performs PCA.

Standard statistical methods were applied to define the primary variables of these networks.

### Data and materials availability

Data has been uploaded to the Metabolomics Workbench (http://www.metabolomicsworkbench.org) with project identifier PR000576.

## Electronic supplementary material

Supplementary Figures and Tables
