# Supplementary material for: Insights into myalgic encephalomyelitis/chronic fatigue syndrome phenotypes through comprehensive metabolomics
Source: Sci Rep. 2018 Jul 3;8:10056. doi: 10.1038/s41598-018-28477-9 (PMC6030047; doi:10.1038/s41598-018-28477-9)
Supplement: Supplementary file 1 — Supplementary Figures and Tables [file 41598_2018_28477_MOESM1_ESM.pdf]

Title: Insights into myalgic encephalomyelitis/chronic fatigue syndrome phenotypes through comprehensive metabolomics

Authors: Dorottya Nagy-Szakal, Dinesh K. Barupal, Bohyun Lee, Xiaoyu Che, Brent L. Williams, Ellie J. R. Kahn, Joy E. Ukaigwe, Lucinda Bateman, Nancy G. Klimas, Anthony L. Komaroff, Susan Levine, Jose G. Montoya, Daniel L. Peterson, Bruce Levin, Mady Hornig, Oliver Fiehn, W. Ian Lipkin

Supplementary Information

The Supplementary Information includes Supplementary Figures and Tables.

Supplementary Materials

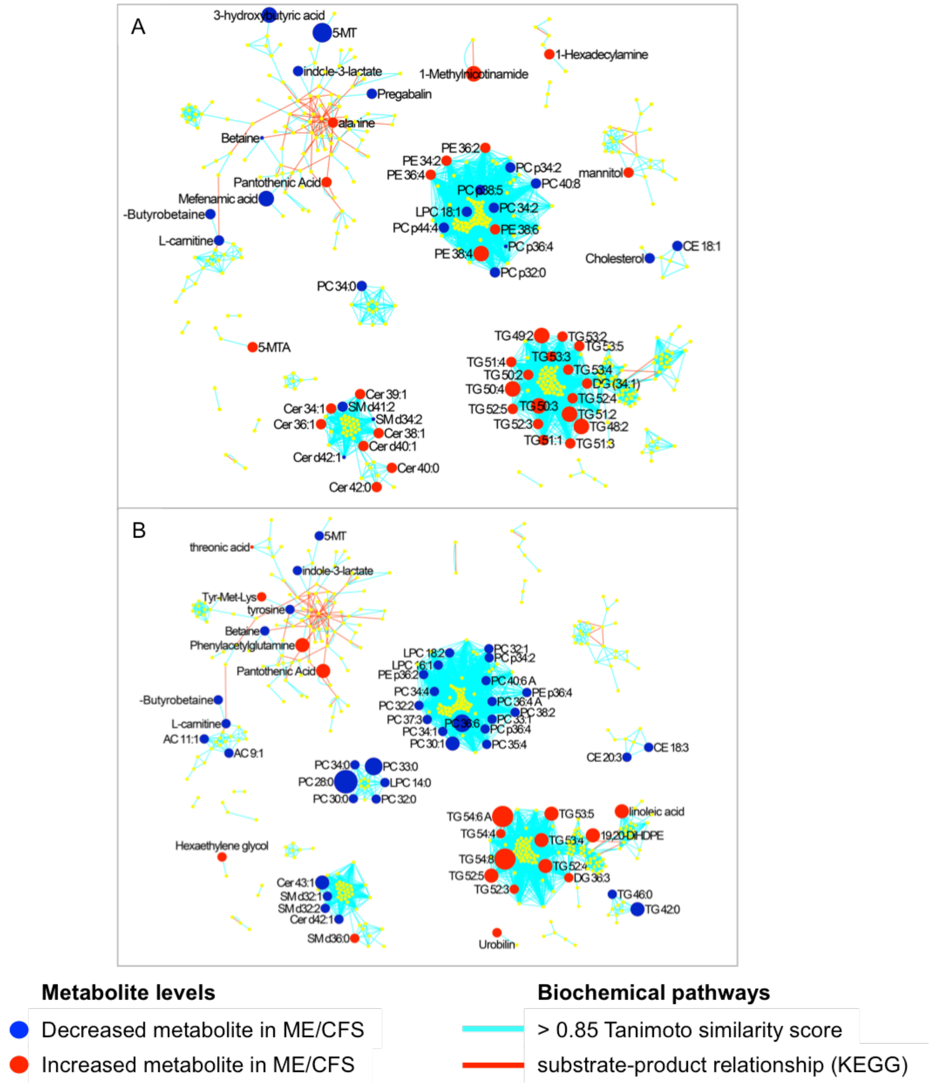

Figure S1. Metabolites differentiate ME/CFS with IBS (A) and without IBS (B) from controls. MetaMapp networks of all identified metabolites in the ME/CFS subgroups were constructed by Tanimoto chemical similarity and KEGG reaction pairs. Each node represents a metabolite. In each node, red indicates up-regulation and blue indicates down-regulation. Node size reflects the magnitude of the effect. Only the compounds that pass a p-value cutoff of 0.05 are labeled. Red lines show the biochemical reactions and blue lines demonstrate chemical similarity scores above 0.85 Tanimoto similarity coefficients. The network was created using www.metamapp.fiehnlab.ucdavis.edu (18) and visualized in the Cytoscape using the organic layout algorithm.

5-MT: 5-methoxytryptamine, 5-MTA: 5-methylthioadenosine, 19,20-DiHDPE: C22H34O4, AC: acylcarnitine, Cer/CE: ceramide, DG: diacylglycerol, FA: fatty acid, LPC: lysophosphatidylcholine, PC: phosphatidylcholine, PE: phosphatidylethanolamine, SM: sphingomyelin, TG: triglyceride, Tyr Met Lys: tyrosine methionine lysine.

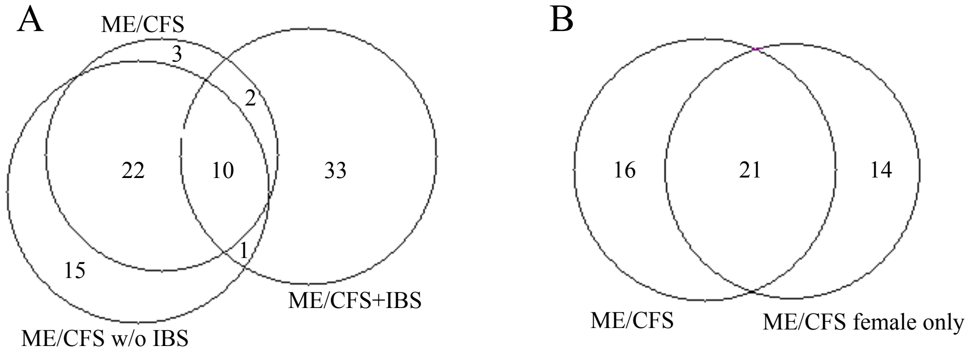

Figure S2. Overlap in ME/CFS metabolic profile subtypes. (A) ME/CFS with IBS has a distinct metabolic profile. A proportional Venn diagram of the number of compounds with p-value < 0.05 in Mann-Whitney U test and logistic regression model (**Table S2 and S3**) shows overlap between all ME/CFS cases and the ME/CFS without IBS subgroup but little overlap with the ME/CFS with IBS subgroup. **(B) The metabolic profiles in women with ME/CFS were similar to those seen in all ME/CFS cases.** The unique and shared metabolites are shown in **Table S4**.

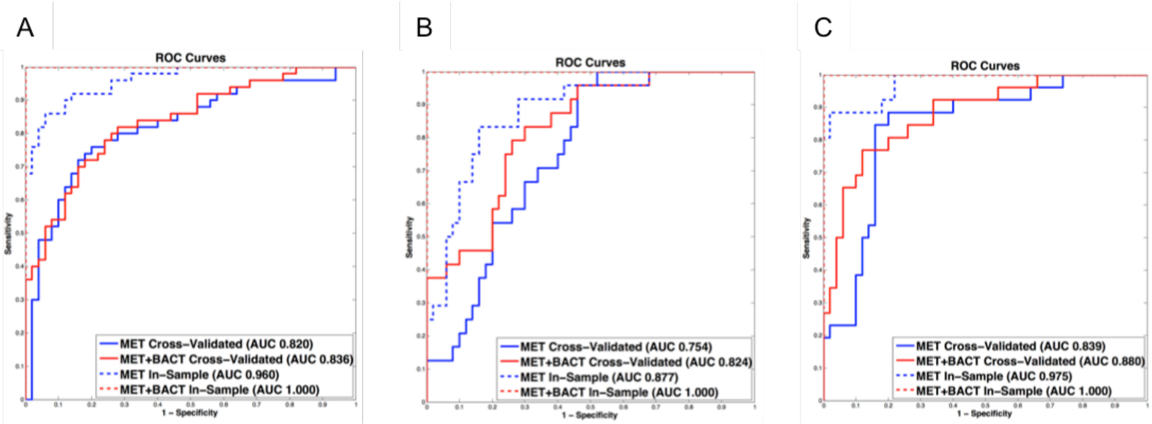

Figure S3. The diagnostic performance (AUROC curves) of ME/CFS and subgroup metabolomics and metagenomics. The Mann-Whitney U test and adjusted univariate logistic regression method were used to screen potential metabolic predictors that differentiate the ME/CFS subgroups from controls, based on all ME/CFS (A) and ME/CFS patients with IBS co-morbidity (B) and without IBS (C). Random forests were used to select top ranked predictors after excluding 5-methoxytryptamine. The top 10 random forests-ranked metabolites were used as predictors in the predictive multivariate logistic regression models (**Tables S2** in ME/CFS, **S3A** in ME/CFS+IBS and **S3B** ME/CFS without IBS). In-sample and cross-validated receiver operating characteristic (ROC) curves were plotted and area under the curve (AUC) was measured to assess the goodness-of-fit and predictive performance of the models. The accuracy of the outcome prediction improved when metagenomic information was added to the models (14). The selected bacterial species are the following: ME/CFS vs. control: *Coprococcus (C.) catus, Pseudoflavonifractor (P.) capillosus, Dorea (D.) formicigenerans, Faecalibacterium (F.) prausnitzii*, *Clostridium (C.) asparigiforme, Sutterella (S.) wadsworthensis, Alistipes (A.) putredinis* and *Anaerotruncus (A.) colihominis*; ME/CFS+IBS vs. control: *F. cf., F. prausnitzii, Bacteroides (B.) vulgatus, A. putredinis, C. catus, Anaerostipes (A.) caccae, D. formicigenerans, A. colihominis* and *C. asparagiforme*; ME/CFS without IBS vs. control: *B. caccae, P. capillosus, Parabacteroides (P.) distasonis, B. fragilis, Prevotella (P.) buccalis, B. xylanisolvens* and *D. formicigenerans.*

Supplementary Tables

Table S1. Exogenous environmental compounds that were excluded. Forty-seven exposomes and vitamins were separately analyzed by Mann-Whitney U test and logistic regression. Green highlighting indicates higher levels in the ME/CFS than the control group. Red indicates lower levels in the ME/CFS group than in the control group. Only pantothenic acid showed significant difference between ME/CFS cases and controls (MWU p=0.014, OR 1.755, p=0.011).

Table S2. Mann-Whitney U test, logistic regression and random forests analyses of metabolites in ME/CFS vs. controls. The nonparametric Mann-Whitney U test and adjusted univariate logistic regression were applied to screen potential metabolic predictors for ME/CFS. Univariate logistic regression adjusted for all frequency matched variables, BMI and IBS. The metabolites with a p-value below 0.05 in both U test and univariate logistic regression were fitted into random forests (17) to develop a representative set of variables. The top 10 random forests-ranked metabolites (highlighted) were used as predictors in the predictive multivariate logistic regression models. 5-methoxytryptamine was not included in random forests analyses as it is confounded by the use of antidepressants in 50% of cases.
ME/CFS: myalgic encephalomyelitis/chronic fatigue syndrome, LPC: lysophosphatidylcholine, TG: triglyceride, PC: phosphatidylcholine, SM: sphingomyelin, CE: ceramide, DG: diacylglycerol, PE: phosphatidylethanolamine, Tyr Met Lys: tyrosine methionine lysine.

Table S3. Mann-Whitney U test, logistic regression and random forests analyses of metabolites in ME/CFS+IBS (A), ME/CFS without IBS (B) and female ME/CFS (C) vs. controls. The nonparametric Mann-Whitney U test and adjusted univariate logistic regression were applied to screen potential metabolic predictors for ME/CFS subgroups. Univariate logistic regression adjusted for all frequency matched variables and BMI. The metabolites with a p-value below 0.05 in both U test and univariate logistic regression were fitted into random forests (17) to develop a representative set of variables. The top 10 random forests-ranked metabolites (highlighted) were used as predictors in the predictive multivariate logistic regression models. 5-methoxytryptamine was not included in random forests analyses as it is confounded by the use of antidepressants in 50% of cases.
ME/CFS: myalgic encephalomyelitis/chronic fatigue syndrome, IBS: irritable bowel syndrome, LPC: lysophosphatidylcholine, TG: triglyceride, PC: phosphatidylcholine, SM: sphingomyelin, CE: ceramide, DG: diacylglycerol, PE: phosphatidylethanolamine, Tyr Met Lys: tyrosine methionine lysine.

Table S4. The unique and shared metabolites in ME/CFS subgroups.

Table S5. Spearman correlation of selected metabolites, bacteria and symptom severity in ME/CFS cases.
Arrows represent the direction of metabolites and bacteria seen in ME/CFS and subgroups compared to controls.

*ME/CFS: myalgic encephalomyelitis/chronic fatigue syndrome, IBS: irritable bowel syndrome, SF-36: Short Form 36 Health Survey, MFI: Multidimensional Fatigue Inventory, TG: triglyceride, PC: phosphatidylcholine, **p<0.01 (Spearman correlation, with positive [red] and negative [blue] correlation).*

Table S6. Clinical symptom assessment in ME/CFS. The table describes the individual surveys with the specific questions listed. Each instrument was transformed into a 0-100 scale to facilitate combination and comparison, wherein a score of 100 is equivalent to maximum disability or severity and a score of zero is equivalent to no disability or disturbance.

Table S7. Median and 25-75% quantiles of chemical compounds differ in ME/CFS and subgroups vs. controls.

Table S1.

Compound name	Direction in ME/CFS	Chemical Pathway	Metabolic Panel	Mann-Whitney U-test p-value	Logistic Regression Odds ratio	Logistic Regression p-value
		ME/CFS vs. Control				
1-hexadecylamine	Decreased	exposome	biogenic amines	0.632	0.837	0.446
2-aminobenzoic acid	Decreased	exposome	biogenic amines	0.410	0.889	0.678
2-hydroxyquinoline	Decreased	exposome	biogenic amines	0.076	0.737	0.303
4-aminophenol B	Increased	exposome	biogenic amines	0.754	1.309	0.349
aniline	Decreased	exposome	biogenic amines	0.770	0.945	0.821
benzoic acid	Increased	exposome	primary metabolites	0.712	1.625	0.285
caffeine	Decreased	exposome	biogenic amines	0.354	0.902	0.676
citral	Increased	exposome	biogenic amines	0.833	1.124	0.724
coniferyl alcohol	Increased	exposome	biogenic amines	0.608	1.109	0.727
cotinine	Decreased	exposome	biogenic amines	0.263	0.948	0.860
cyclohexanamine	Increased	exposome	biogenic amines	0.372	1.728	0.167
D panthenol	Increased	exposome	biogenic amines	0.723	1.164	0.423
dehydroascorbic acid	Increased	exposome	primary metabolites	0.372	1.115	0.638
diatrizoic acid	Decreased	exposome	biogenic amines	0.211	0.558	0.117
diaveridine	Increased	exposome	biogenic amines	0.347	1.540	0.110
dihydrocoumarin	Decreased	exposome	biogenic amines	0.579	0.983	0.944
dimethyl sulfoxide	Decreased	exposome	biogenic amines	0.775	0.772	0.328
dioctyl phthalate	Decreased	exposome	biogenic amines	0.915	0.904	0.738
dodecanol	Increased	exposome	primary metabolites	0.893	1.184	0.531
hexaethylene glycol	Increased	exposome	biogenic amines	0.091	1.639	0.029
hippuric acid	Increased	exposome	biogenic amines	0.764	1.054	0.844
hippuric acid	Decreased	exposome	primary metabolites	0.519	0.915	0.716
ibuprofen	Increased	exposome	primary metabolites	0.828	1.334	0.072
imazamethabenz methyl	Increased	exposome	biogenic amines	0.180	1.768	0.118
mefenamic acid	Decreased	exposome	biogenic amines	0.037	0.705	0.165
methyl cinnamate	Increased	exposome	biogenic amines	0.038	2.522	0.203
methyljasmonate	Decreased	exposome	biogenic amines	0.812	0.723	0.078
minoxidil	Decreased	exposome	biogenic amines	0.707	0.761	0.404
myosmine	Increased	exposome	biogenic amines	0.738	1.097	0.734
N-3-aminomethyl-benzyl-acetamidine	Decreased	exposome	biogenic amines	0.593	0.861	0.592
N-cyclohexylformamide	Increased	exposome	biogenic amines	0.361	1.554	0.155
N-Tris-hydroxymethyl-methyl-4-aminobutanesulfonic acid	Increased	exposome	biogenic amines	0.149	1.792	0.178
naproxen	Decreased	exposome	primary metabolites	0.368	0.790	0.463
nicotinamide	Increased	vitamin	biogenic amines	0.039	1.372	0.088
pantothenic acid	Increased	vitamin	biogenic amines	**0.014**	**1.755**	**0.011**
phenol	Decreased	exposome	primary metabolites	0.692	0.989	0.968
pinene oxide	Increased	exposome	biogenic amines	0.476	1.047	0.857
piperine	Decreased	exposome	biogenic amines	0.687	0.919	0.707
pregabalin	Decreased	exposome	biogenic amines	0.062	0.796	0.348
quinic acid	Decreased	exposome	primary metabolites	0.189	0.811	0.377
salicylic acid	Decreased	exposome	primary metabolites	0.329	0.421	0.108
tetradecylamine	Decreased	exposome	biogenic amines	0.764	0.910	0.686
tetraethylene glycol monomethyl ether	Increased	exposome	biogenic amines	0.281	1.606	0.166
tetrapentylammonium cation	Increased	exposome	biogenic amines	0.326	1.185	0.507
theobromine	Decreased	exposome	biogenic amines	0.637	0.906	0.643
tocopherol alpha	Decreased	vitamin	primary metabolites	0.372	0.956	0.841
triethyl citrate	Increased	exposome	biogenic amines	0.749	1.038	0.891

Table S2.

ME/CFS vs. Control								
Compound name	Direction in ME/CFS	Chemical Pathway	Metabolic Panel	Mann-Whitney U-test p-value	Logistic Regression		Random Forest	
					Odds ratio	p-value	Importance	RF ranking
LPC 18:2	Decreased	PC	complex lipids	0.004	0.512	0.013	0.629	1
Betaine	Decreased	carnitine-choline	biogenic amines	0.006	0.551	0.021	0.537	2
TG 53:5	Increased	TG	complex lipids	0.001	1.699	0.028	0.422	3
α N-phenylacetyl-L-glutamine	Increased	amino acid metabolism	biogenic amines	0.004	2.457	0.015	0.376	4
PC 30:0	Decreased	PC	complex lipids	0.017	0.330	0.001	0.352	5
SM d32:1	Decreased	SM	complex lipids	0.017	0.513	0.009	0.346	6
PC 33:0	Decreased	PC	complex lipids	0.002	0.397	0.000	0.304	7
Urobilin	Increased	energy	biogenic amines	0.010	2.086	0.023	0.283	8
ε Caprolactam	Increased	amino acid	biogenic amines	0.033	1.925	0.041	0.276	9
TG 54:8	Increased	TG	complex lipids	0.003	1.751	0.018	0.257	10
γ Butyrobetaine	Decreased	carnitine-choline	biogenic amines	0.001	0.599	0.027	0.248	11
PC p34:2 or PC o34:3	Decreased	PC	complex lipids	0.027	0.549	0.033	0.230	12
TG 53:4	Increased	TG	complex lipids	0.002	1.896	0.025	0.222	13
L-carnitine	Decreased	carnitine-choline	biogenic amines	0.005	0.378	0.003	0.190	14
TG 52:5	Increased	TG	complex lipids	0.002	1.994	0.034	0.173	15
PC 28:0	Decreased	PC	complex lipids	0.006	0.373	0.001	0.166	16
PC 34:0	Decreased	PC	complex lipids	0.008	0.513	0.015	0.165	17
TG 54:6 A	Increased	TG	complex lipids	0.015	2.222	0.007	0.157	18
PC 36:4 A	Decreased	PC	complex lipids	0.050	0.569	0.037	0.156	19
Indole-3-lactate	Decreased	neurotransmitter	primary metabolites	0.007	0.549	0.023	0.151	20
Tyr Met Lys	Increased	amino acid	biogenic amines	0.025	2.801	0.012	0.119	21
TG 52:4	Increased	TG	complex lipids	0.002	2.438	0.007	0.118	22
TG 52:3	Increased	TG	complex lipids	0.005	2.048	0.023	0.088	23
PC 34:4	Decreased	PC	complex lipids	0.032	0.511	0.007	0.081	24
LPC 16:1	Decreased	PC	complex lipids	0.024	0.389	0.004	0.078	25
Acylcarnitine C9:1	Decreased	carnitine	biogenic amines	0.022	0.502	0.025	0.065	26
PC 32:2	Decreased	PC	complex lipids	0.012	0.478	0.004	0.058	27
PC 32:0	Decreased	PC	complex lipids	0.006	0.415	0.010	0.051	28
PC p36:4 or PC o36:5	Decreased	PC	complex lipids	0.024	0.537	0.022	0.038	29
CE 18:3	Decreased	cholesterol	complex lipids	0.024	0.598	0.018	0.025	30
PC 35:4	Decreased	PC	complex lipids	0.017	0.521	0.011	0.012	31
PC 34:1	Decreased	PC	complex lipids	0.012	0.427	0.010	0.001	32
TG 54:5 A	Increased	TG	complex lipids	0.021	1.741	0.040	-0.025	33
DG 36:3	Increased	mono-, diacylglyceride	complex lipids	0.024	1.954	0.014	-0.032	34
Acylcarnitine C11:1	Decreased	carnitines	biogenic amines	0.025	0.599	0.027	-0.033	35
PE p36:4 or PE o36:5	Decreased	PE	complex lipids	0.033	0.442	0.013	-0.045	36
5-methoxytryptamine	Decreased	neurotransmitter	primary metabolites	0.005	0.643	0.036		

Table S3.

A. ME/CFS+IBS vs. Control								
Compound name	Direction in ME/CFS+IBS	Chemical Pathway	Metabolic Panel	Mann-Whitney U-test p-value	Logistic Regression		Random Forest	
					Odds ratio	p-value	Importance	RF ranking
LPC 18:2	Decreased	PC	complex lipids	0.000	0.305	0.004	1.475	1
Ceramide d36:1	Increased	ceramide	complex lipids	0.002	2.825	0.015	1.028	2
γ Butyrobetaine	Decreased	carnitine-choline	biogenic amines	0.000	0.470	0.003	0.827	3
LPC 18:1	Decreased	PC	complex lipids	0.001	0.396	0.011	0.751	4
5-Methylthioadenosine	Increased	one-carbon / nicotinate	biogenic amines	0.001	3.715	0.005	0.706	5
TG 49:2	Increased	TG	complex lipids	0.006	1.844	0.043	0.618	6
Ceramide d40:0	Increased	ceramide	complex lipids	0.002	2.340	0.024	0.499	7
TG 51:3	Increased	TG	complex lipids	0.001	2.459	0.020	0.496	8
Betaine	Decreased	carnitine-choline	biogenic amines	0.016	0.555	0.028	0.381	9
Ceramide d42:0	Increased	ceramide	complex lipids	0.016	2.014	0.028	0.365	10
LPC p16:0 or LPC o16:1	Decreased	PC	complex lipids	0.001	0.483	0.026	0.357	11
Ceramide d34:1	Increased	ceramide	complex lipids	0.005	2.257	0.025	0.288	12
Indole-3-lactate	Decreased	neurotransmitter	primary metabolites	0.020	0.484	0.032	0.286	13
TG 50:3	Increased	TG	complex lipids	0.003	2.214	0.017	0.253	14
TG 53:4	Increased	TG	complex lipids	0.003	2.807	0.007	0.235	15
PE 38:6	Increased	PE	complex lipids	0.009	6.836	0.012	0.146	16
TG 53:3	Increased	TG	complex lipids	0.014	3.026	0.013	0.125	17
TG 51:4	Increased	TG	complex lipids	0.017	2.020	0.029	0.114	18
TG 52:5	Increased	TG	complex lipids	0.013	2.718	0.019	0.107	19
PC 36:4 A	Decreased	PC	complex lipids	0.021	0.512	0.036	0.099	20
TG 53:5	Increased	TG	complex lipids	0.008	2.159	0.016	0.098	21
TG 51:2	Increased	TG	complex lipids	0.006	2.140	0.020	0.083	22
TG 50:4	Increased	TG	complex lipids	0.004	2.614	0.009	0.075	23
DG 34:1	Increased	mono-, diacylglyceride	complex lipids	0.042	2.427	0.040	0.073	24
Mannitol	Increased	sugar alcohol	primary metabolites	0.012	1.998	0.043	0.073	25
PC 34:2	Decreased	PC	complex lipids	0.009	0.429	0.009	0.039	26
Ceramide d38:1	Increased	ceramide	complex lipids	0.004	2.961	0.005	0.035	27
PC p32:0 or PC o32:1	Decreased	PC	complex lipids	0.011	0.519	0.024	0.034	28
PC p34:2 or PC o34:3	Decreased	PC	complex lipids	0.028	0.467	0.014	0.030	29
TG 51:1	Increased	TG	complex lipids	0.042	1.792	0.038	0.023	30
TG 53:2	Increased	TG	complex lipids	0.025	2.281	0.022	0.020	31
CE 18:1	Decreased	cholesterol	complex lipids	0.013	0.487	0.024	0.020	32
PE 34:2	Increased	PE	complex lipids	0.013	2.184	0.028	0.019	33
TG 52:4	Increased	TG	complex lipids	0.038	2.184	0.027	0.017	34
L-alanine	Increased	amino acid	biogenic amines	0.019	1.883	0.048	0.005	35
L-carnitine	Decreased	carnitine-choline	biogenic amines	0.035	0.554	0.041	0.004	36
PE 38:4 B	Increased	PE	complex lipids	0.011	1.993	0.037	0.004	37
TG 50:2	Increased	TG	complex lipids	0.031	1.811	0.046	0.002	38
PC 40:8	Decreased	PC	complex lipids	0.038	0.576	0.050	-0.001	39
PE 36:4	Increased	PE	complex lipids	0.028	1.752	0.049	-0.003	40
Cholesterol	Increased	cholesterol	primary metabolites	0.044	0.557	0.034	-0.005	41
Ceramide d40:1	Increased	ceramide	complex lipids	0.048	2.490	0.018	-0.021	42
PE 36:2	Increased	PE	complex lipids	0.042	2.015	0.035	-0.033	43
TG 48:2	Increased	TG	complex lipids	0.037	1.878	0.039	-0.080	44
5-methoxytryptamine	Decreased	neurotransmitter	primary metabolites	0.005	0.450	0.002		

B. ME/CFS w/o IBS vs. Control								
**Compound name**	**Direction in ME/CFS w/o IBS**	**Chemical Pathway**	**Metabolic Panel**	**Mann-Whitney U-test p-value**	**Logistic Regression**		**Random Forest**	
					**Odds ratio**	**p-value**	**Importance**	**RF ranking**
PC 33:0	Decreased	PC	complex lipids	0.000	0.386	0.000	1.309	1
PC 38:2	Decreased	PC	complex lipids	0.000	0.487	0.007	0.817	2
PC 30:0	Decreased	PC	complex lipids	0.000	0.323	0.001	0.599	3
tyrosine	Decreased	amino acid	primary metabolites	0.008	0.333	0.001	0.590	4
PC 36:6	Decreased	PC	complex lipids	0.028	0.545	0.015	0.427	5
TG 54:6 A	Increased	TG	complex lipids	0.001	2.267	0.009	0.392	6
TG 53:5	Increased	TG	complex lipids	0.004	1.736	0.030	0.351	7
TG 54:8	Increased	TG	complex lipids	0.003	1.674	0.030	0.335	8
Tyr Met Lys	Increased	amino acid	biogenic amines	0.011	2.893	0.015	0.312	9
PC 32:2	Decreased	PC	complex lipids	0.001	0.483	0.006	0.312	10
PC 32:1	Decreased	PC	complex lipids	0.002	0.342	0.004	0.266	11
LPC 18:2	Decreased	PC	complex lipids	0.008	0.530	0.017	0.239	12
LPC 16:1	Decreased	PC	complex lipids	0.003	0.418	0.007	0.233	13
PC 32:1	Decreased	PC	complex lipids	0.012	0.518	0.009	0.224	14
SM d32:1	Decreased	SM	complex lipids	0.005	0.500	0.009	0.216	15
PC 28:0	Decreased	PC	complex lipids	0.001	0.378	0.001	0.210	16
PC 34:4	Decreased	PC	complex lipids	0.003	0.504	0.008	0.205	17
Linoleic acid	Increased	fatty acid	primary metabolites	0.035	1.781	0.027	0.174	18
SM d32:2	Decreased	SM	complex lipids	0.015	0.561	0.021	0.161	19
PC 32:0	Decreased	PC	complex lipids	0.004	0.387	0.010	0.160	20
Acylcarnitine C9:1	Decreased	carnitine	biogenic amines	0.009	0.478	0.024	0.152	21
TG 52:3	Increased	TG	complex lipids	0.004	2.102	0.023	0.145	22
PC p34:2 or PC o34:3	Decreased	PC	complex lipids	0.043	0.542	0.035	0.132	23
Indole-3-lactate	Decreased	neurotransmitter	primary metabolites	0.035	0.545	0.024	0.129	24
PC 34:0	Decreased	PC	complex lipids	0.012	0.488	0.014	0.124	25
TG 54:4	Increased	TG	complex lipids	0.009	1.986	0.027	0.117	26
TG 52:4	Increased	TG	complex lipids	0.003	2.422	0.009	0.115	27
TG 52:5	Increased	TG	complex lipids	0.011	1.930	0.045	0.107	28
PC p36:4 or PC o36:5	Decreased	PC	complex lipids	0.045	0.512	0.018	0.103	29
DG 36:3	Increased	mono-, diacylglyceride	complex lipids	0.010	1.960	0.017	0.097	30
TG 14:0:14:0:14:0	Decreased	TG	complex lipids	0.031	0.418	0.032	0.095	31
PC 34:4	Decreased	PC	complex lipids	0.008	0.471	0.005	0.094	32
Urobilin	Increased	energy	biogenic amines	0.016	2.408	0.017	0.091	33
L-carnitine	Decreased	carnitine-choline	biogenic amines	0.016	0.352	0.002	0.091	34
PC 32:2	Decreased	PC	complex lipids	0.015	0.559	0.014	0.075	35
PC 32:0	Decreased	PC	complex lipids	0.025	0.594	0.038	0.074	36
PC 33:1	Decreased	PC	complex lipids	0.012	0.544	0.024	0.065	37
Betaine	Decreased	carnitine-choline	biogenic amines	0.040	0.548	0.025	0.064	38
PC 40:6 A	Decreased	PC	complex lipids	0.030	0.525	0.022	0.062	39
PC 37:3	Decreased	PC	complex lipids	0.007	0.560	0.020	0.058	40
Ceramide d43:1	Decreased	ceramide	complex lipids	0.007	0.469	0.008	0.050	41
Ceramide d42:1	Decreased	ceramide	complex lipids	0.035	0.600	0.050	0.042	42
PE p36:4 or PE o36:5	Decreased	PE	complex lipids	0.033	0.411	0.011	0.026	43
PC 36:4 A	Decreased	PC	complex lipids	0.031	0.567	0.039	0.025	44
PC 30:1	Decreased	PC	complex lipids	0.015	0.543	0.032	0.025	45
PC 35:4	Decreased	PC	complex lipids	0.021	0.504	0.010	0.023	46
LPC 14:0	Decreased	PC	complex lipids	0.010	0.482	0.009	0.021	47
Acylcarnitine C11:1	Decreased	carnitine	biogenic amines	0.016	0.613	0.036	0.016	48
PC 34:1	Decreased	PC	complex lipids	0.007	0.435	0.013	0.012	49
TG 53:4	Increased	TG	complex lipids	0.036	1.891	0.030	0.008	50
CE 18:3	Decreased	cholesterol	complex lipids	0.020	0.574	0.017	0.005	51
PC 38:2	Decreased	PC	complex lipids	0.025	0.600	0.049	-0.004	52

C. ME/CFS vs. Control -female only								
**Compound name**	**Direction in ME/CFS**	**Chemical Pathway**	**Metabolic Panel**	**Mann-Whitney U-test p-value**	**Logistic Regression**		**Random Forest**	
					**Odds ratio**	**p-value**	**Importance**	**RF ranking**
LPC 18:2	Decreased	PC	complex lipids	0.0002	0.3681	0.0107	1.2410	1
TG 53:5	Increased	TG	complex lipids	0.0001	2.9681	0.0018	0.9402	2
TG 51:3	Increased	TG	complex lipids	0.0009	2.0010	0.0486	0.9080	3
indole-3-lactate	Decreased	neurotransmitter	primary metabolites	0.0010	0.3698	0.0051	0.7401	4
LPC 18:2	Decreased	PC	complex lipids	0.0020	0.3632	0.0058	0.7082	5
TG 52:3	Increased	TG	complex lipids	0.0002	3.4103	0.0044	0.5521	6
PC 30:0	Decreased	PC	complex lipids	0.0236	0.2204	0.0013	0.5184	7
γ-butyrobetaine	Decreased	carnitine-choline	biogenic amines	0.0004	0.5708	0.0222	0.5139	8
PC 28:0	Decreased	PC	complex lipids	0.0036	0.2859	0.0005	0.4149	9
PC 33:0	Decreased	PC	complex lipids	0.0048	0.3603	0.0003	0.3583	10
LPC 20:2	Decreased	PC	complex lipids	0.0068	0.5392	0.0490	0.3559	11
TG 52:5	Increased	TG	complex lipids	0.0008	2.6205	0.0253	0.3218	12
LPC 18:1	Decreased	PC	complex lipids	0.0017	0.4218	0.0266	0.3019	13
TG 52:4	Increased	TG	complex lipids	0.0003	4.4958	0.0017	0.2485	14
LPE 18:2	Decreased	PE	complex lipids	0.0129	0.4394	0.0151	0.2147	15
L-carnitine	Decreased	carnitine-choline	biogenic amines	0.0052	0.4020	0.0119	0.2011	16
TG 53:3	Increased	TG	complex lipids	0.0016	2.6514	0.0167	0.1841	17
CE 18:3	Decreased	cholesterol	complex lipids	0.0155	0.5808	0.0233	0.1791	18
SM d32:1	Decreased	SM	complex lipids	0.0493	0.5227	0.0352	0.1507	19
PC 32:0	Decreased	PC	complex lipids	0.0021	0.2406	0.0037	0.1302	20
PC 34:0	Decreased	PC	complex lipids	0.0054	0.4848	0.0155	0.1150	21
TG 51:4	Increased	TG	complex lipids	0.0016	2.4861	0.0079	0.1133	22
PC 40:8	Decreased	PC	complex lipids	0.0076	0.4818	0.0475	0.1101	23
TG 53:4	Increased	TG	complex lipids	0.0006	2.8004	0.0064	0.1094	24
TG 54:5 A	Increased	TG	complex lipids	0.0062	3.9854	0.0028	0.1092	25
leucine	Decreased	amino acid	biogenic amines	0.0123	0.4691	0.0403	0.1064	26
PC p-36:4 or PC o-36:5	Decreased	PC	complex lipids	0.0300	0.4340	0.0187	0.1013	27
DG 36:3	Increased	DG	complex lipids	0.0054	2.8206	0.0037	0.0926	28
TG 54:6 A	Increased	TG	complex lipids	0.0194	3.9758	0.0043	0.0791	29
LPC 16:1	Decreased	PC	complex lipids	0.0180	0.2910	0.0037	0.0678	30
PC p-34:2 or PC o-34:3	Decreased	PC	complex lipids	0.0194	0.4172	0.0118	0.0674	31
PC 35:4	Decreased	PC	complex lipids	0.0329	0.5079	0.0209	0.0564	32
PC 34:1	Decreased	PC	complex lipids	0.0113	0.1712	0.0037	0.0551	33
PC p-34:2 or PC o-34:3	Decreased	PC	complex lipids	0.0129	0.5135	0.0317	0.0367	34
LPC 16:0	Decreased	PC	complex lipids	0.0209	0.4578	0.0278	0.0116	35
Acylcarnitine C9:1	Decreased	carnitine	biogenic amines	0.0286	0.3774	0.0211	0.0053	36
LPC 18:1	Decreased	PC	complex lipids	0.0293	0.4576	0.0281	0.0029	37
PC p-36:4 or PC o-36:5	Decreased	PC	complex lipids	0.0280	0.4865	0.0233	-0.0059	38
PE p-36:4 or PE o-36:5	Decreased	PE	complex lipids	0.0236	0.3779	0.0125	-0.0094	39

Table S4.

(A) ME/CFS and IBS co-morbidity			
Unique metabolites		Shared metabolites	
all ME/CFS	α N-phenylacetyl-L-glutamine	in all group	Betaine
3	TG 54:5 A	10	PC 36:4 A
	ε Caprolactam		TG 52:5
ME/CFS+IBS all ME/CFS	γ Butyrobetaine		LPC 18:2
2	5-methoxytryptamine		Indole-3-lactate
ME/CFS w/o IBS all ME/CFS	CE 18:3		PC p34:2 or PC o34:3
22	PC 30:0		L-carnitine
	PC 34:4		TG 52:4
	LPC 16:1		TG 53:5
	SM d32:1		TG 53:4
	Acylcarnitine C9:1	Shared between all ME/CFS and ME/CFS+IBS	γ Butyrobetaine
	DG 36:3	2	5-methoxytryptamine
	TG 54:8	Shared between all ME/CFS and ME/CFS w/o IBS	CE 18:3
	PC 32:2	22	PC 30:0
	PC 35:4		PC 34:4
	Urobilin		LPC 16:1
	Tyr Met Lys		SM d32:1
	PC p36:4 or PC o36:5		Acylcarnitine C9:1
	TG 54:6 A		DG 36:3
	PE p36:4 or PE o36:5		TG 54:8
	PC 33:0		PC 32:2
	PC 32:0		PC 35:4
	PC 28:0		Urobilin
	PC 34:1		Tyr Met Lys
	PC 34:0		PC p36:4 or PC o36:5
	TG 52:3		TG 54:6 A
	Acylcarnitine C11:1		PE p36:4 or PE o36:5
			PC 33:0
			PC 32:0
			PC 28:0
			PC 34:1
			PC 34:0
			TG 52:3
			Acylcarnitine C11:1

(B) ME/CFS and ME/CFS female only			
Unique metabolites		Shared metabolites	
all ME/CFS	5-methoxytryptamine	Shared between ME/CFS female only all ME/CFS	Acylcarnitine C9:1
16	Acylcarnitine C11:1	21	CE 18:3
	Betaine		DG 36:3
	α N-phenylacetyl-L-glutamine		L-carnitine
	γ Butyrobetaine		LPC 16:1
	Indole-3-lactate		LPC 18:2
	ε Caprolactam		PC 28:0
	PC 32:2		PC 30:0
	PC 34:4		PC 32:0
	PC 36:4 A		PC 33:0
	PC p34:2 or PC o34:3		PC 34:0
	PC p36:4 or PC o36:5		PC 34:1
	PE p36:4 or PE o36:5		PC 35:4
	TG 54:8		SM d32:1
	Tyr Met Lys		TG 52:3
	Urobilin		TG 52:4
ME/CFS female only	LPE 18:2		TG 52:5
14	γ Butyrobetaine		TG 53:4
	indole-3-lactate		TG 53:5
	leucine		TG 54:5 A
	LPC 16:0		TG 54:6 A
	LPC 18:1		
	LPC 20:2		
	PC 40:8		
	PC p-34:2 or PC o-34:3		
	PC p-36:4 or PC o-36:5		
	PE p-36:4 or PE o-36:5		
	TG 51:3		
	TG 51:4		
	TG 53:3		

Table S5.

ME/CFS cases (n=50)		Betaine	PC 30:0
		↓	↓
[Bacteroidetes] Alistipes putredinis	↓		0.426**
[Firmicutes] Anaerotruncus colihominis	↑	-0.414**	

ME/CFS+IBS cases (n=24)		γ Butyrobetaine	5-Methylthio-adenosine	Ceramide d42:0
		↓	↑	↑
[Firmicutes] Faecalibacterium cf	↓	-0.558**		
SF-36 General health			0.557**	
MFI Physical fatigue				0.516**

ME/CFS without IBS cases (n=26)		Tyrosine	TG 54:6 A	TG 54:8
		↓	↑	↑
[Bacteroidetes] Bacteroides caccae	↓			-0.530**
[Bacteroidetes] Parabacteroides distasonis	↓	0.498**		
[Firmicutes] Dorea formicigenerans	↓		-0.515**	-0.574**

Table S6.

**Questionnaire**	**Assessment**		**Questions used**	**Reference**
Cognitive function	DePaul Symptom Questionnaire (DSQ) to assess cognitive function	DSQ	24 25 26 27 28 31 32	*Jason et al. Am J Biochem Biotechnol (2010)*
	Modified Cognitive Disturbance	DSQ	4 24 25 26 27 28 31 32	
Sleeping	DePaul Symptom Questionnaire (DSQ) to assess sleep disturbances	DSQ	7 8 9 10 11 12	
	Pittsburgh Sleep Quality Index (PSQI) to assess sleep disturbances	PSQI	1 2 3 4 5 6 7 8 9 10	*Buysse et al. Psychiat Res (1989)*
Short Form 36 Health Survey (SF-36)	PHYSICAL FUNCTION	SF-36	3 4 5 6 7 8 9 10 11 12	*Ware et al. Med Care (1992)*
	PHYSICAL LIMITATION	SF-36	13 14 15 16	
	ENERGY SUBSCALE	SF-36	23 27 29 31	
	EMOTIONAL LIMITATION	SF-36	17 18 19	
	EMOTIONAL WELLBEING	SF-36	24 25 26 28 30	
	SOCIAL FUNCTION	SF-36	20 32	
	PAIN SUBSCALE	SF-36	21 22	
	GENERAL SUBSCALE/HEALTH	SF-36	1 33 34 35 36	
	HEALTH CHANGE	SF-36	2	
Multidimensional Fatigue Inventory (MFI)	GENERAL FATIGUE	MFI (20)	1 5 12 16	*Smets et al. J Psychosom Res (1995)*
	PHYSICAL FATIGUE	MFI (20)	2 8 14 20	
	REDUCED ACTIVITY	MFI (20)	3 6 10 17	
	MENTAL FATIGUE	MFI (20)	7 11 13 19	
	REDUCED MOTIVATION	MFI (20)	4 9 15 18	

Specific questions

DSQ

1. Fatigue/extreme tiredness: Did you have this symptom BEFORE CFS?
Over the PAST 6 MONTHS, how OFTEN have you had this symptom?: Fatigue/extreme tiredness
Over the PAST 6 MONTHS, how MUCH has this symptom bothered you?: Fatigue/extreme tiredness
2. Dead, heavy feeling after starting to exercise: Did you have this symptom BEFORE CFS?
Over the PAST 6 MONTHS, how OFTEN have you had this symptom?: Dead, heavy feeling after starting to exercise
Over the PAST 6 MONTHS, how MUCH has this symptom bothered you?: Dead, heavy feeling after starting to exercise
3. Next day soreness or fatigue after non-strenuous, everyday activities: Did you have this symptom BEFORE CFS?
Over the PAST 6 MONTHS, how OFTEN have you had this symptom?: Next day soreness or fatigue after non-strenuous, everyday activities
Over the PAST 6 MONTHS, how MUCH has this symptom bothered you?:Next day soreness or fatigue after non-strenuous, everyday activities
4. Mentally tired after the slightest effort: Did you have this symptom BEFORE CFS?
Over the PAST 6 MONTHS, how OFTEN have you had this symptom?: Mentally tired after the slightest effort
Over the PAST 6 MONTHS, how MUCH has this symptom bothered you?:Mentally tired after the slightest effort
5. Minimum exercise makes you physically tired: Did you have this symptom BEFORE CFS?
Over the PAST 6 MONTHS, how OFTEN have you had this symptom?: Minimum exercise makes you physically tired
Over the PAST 6 MONTHS, how MUCH has this symptom bothered you?: Minimum exercise makes you physically tired
6. Physically drained or sick after mild activity: Did you have this symptom BEFORE CFS?
Over the PAST 6 MONTHS, how OFTEN have you had this symptom?: Physically drained or sick after mild activity
Over the PAST 6 MONTHS, how MUCH has this symptom bothered you?: Physically drained or sick after mild activity
7. Feeling unrefreshed after you wake up in the morning: Did you have this symptom BEFORE CFS?
Over the PAST 6 MONTHS, how OFTEN have you had this symptom?: Feeling unrefreshed after you wake up in the morning
Over the PAST 6 MONTHS, how MUCH has this symptom bothered you?: Feeling unrefreshed after you wake up in the morning
8. Need to nap daily: Did you have this symptom BEFORE CFS?
Over the PAST 6 MONTHS, how OFTEN have you had this symptom?: Need to nap daily
Over the PAST 6 MONTHS, how MUCH has this symptom bothered you?: Need to nap daily
9. Problems falling asleep: Did you have this symptom BEFORE CFS?
Over the PAST 6 MONTHS, how OFTEN have you had this symptom?: Problems falling asleep
Over the PAST 6 MONTHS, how MUCH has this symptom bothered you?: Problems falling asleep
10. Problems staying asleep: Did you have this symptom BEFORE CFS?
Over the PAST 6 MONTHS, how OFTEN have you had this symptom?: Problems staying asleep
Over the PAST 6 MONTHS, how MUCH has this symptom bothered you?: Problems staying asleep
11. Waking up early in the morning (e.g. 3am): Did you have this symptom BEFORE CFS?
Over the PAST 6 MONTHS, how OFTEN have you had this symptom?: Waking up early in the morning (e.g. 3am)
Over the PAST 6 MONTHS, how MUCH has this symptom bothered you?: Waking up early in the morning (e.g. 3am)
12. Sleep all day and stay awake all night: Did you have this symptom BEFORE CFS?
Over the PAST 6 MONTHS, how OFTEN have you had this symptom?: Sleep all day and stay awake all night
Over the PAST 6 MONTHS, how MUCH has this symptom bothered you?: Sleep all day and stay awake all night
13. Pain or aching in your muscles: Did you have this symptom BEFORE CFS?
Over the PAST 6 MONTHS, how OFTEN have you had this symptom?: Pain or aching in your muscles
Over the PAST 6 MONTHS, how MUCH has this symptom bothered you?: Pain or aching in your muscles
14. Pain/stiffness/tenderness in more than one joint without swelling or redness: Did you have this symptom BEFORE CFS?
Over the PAST 6 MONTHS, how OFTEN have you had this symptom?: Pain/stiffness/tenderness in more than one joint without swelling or redness
Over the PAST 6 MONTHS, how MUCH has this symptom bothered you?: Pain/stiffness/tenderness in more than one joint without swelling or redness
15. Eye pain: Did you have this symptom BEFORE CFS?
Over the PAST 6 MONTHS, how OFTEN have you had this symptom?: Eye pain
Over the PAST 6 MONTHS, how MUCH has this symptom bothered you?: Eye pain
16. Chest pain: Did you have this symptom BEFORE CFS?
Over the PAST 6 MONTHS, how OFTEN have you had this symptom?: Chest pain
Over the PAST 6 MONTHS, how MUCH has this symptom bothered you?: Chest pain
17. Bloating: Did you have this symptom BEFORE CFS?
Over the PAST 6 MONTHS, how OFTEN have you had this symptom?: Bloating
Over the PAST 6 MONTHS, how MUCH has this symptom bothered you?: Bloating
18. Abdomen/stomach pain: Did you have this symptom BEFORE CFS?
Over the PAST 6 MONTHS, how OFTEN have you had this symptom?: Abdomen/stomach pain
Over the PAST 6 MONTHS, how MUCH has this symptom bothered you?: Abdomen/stomach pain
19. Headaches: Did you have this symptom BEFORE CFS?
Over the PAST 6 MONTHS, how OFTEN have you had this symptom?: Headaches
Over the PAST 6 MONTHS, how MUCH has this symptom bothered you?: Headaches
20. Muscle twitches: Did you have this symptom BEFORE CFS?
Over the PAST 6 MONTHS, how OFTEN have you had this symptom?: Muscle twitches
Over the PAST 6 MONTHS, how MUCH has this symptom bothered you?: Muscle twitches
21. Muscle weakness: Did you have this symptom BEFORE CFS?
Over the PAST 6 MONTHS, how OFTEN have you had this symptom?: Muscle weakness
Over the PAST 6 MONTHS, how MUCH has this symptom bothered you?: Muscle weakness
22. Sensitivity to noise: Did you have this symptom BEFORE CFS?
Over the PAST 6 MONTHS, how OFTEN have you had this symptom?: Sensitivity to noise
Over the PAST 6 MONTHS, how MUCH has this symptom bothered you?: Sensitivity to noise
23. Sensitivity to bright lights: Did you have this symptom BEFORE CFS?
Over the PAST 6 MONTHS, how OFTEN have you had this symptom?: Sensitivity to bright lights
Over the PAST 6 MONTHS, how MUCH has this symptom bothered you?: Sensitivity to bright lights
24. Problems remembering things: Did you have this symptom BEFORE CFS?
Over the PAST 6 MONTHS, how OFTEN have you had this symptom?: Problems remembering things
Over the PAST 6 MONTHS, how MUCH has this symptom bothered you?: Problems remembering things
25. Difficulty paying attention: Did you have this symptom BEFORE CFS?
Over the PAST 6 MONTHS, how OFTEN have you had this symptom?: Difficulty paying attention
Over the PAST 6 MONTHS, how MUCH has this symptom bothered you?: Difficulty paying attention
26. Difficulty finding the right word to say or expressing thoughts: Did you have this symptom BEFORE CFS?
Over the PAST 6 MONTHS, how OFTEN have you had this symptom?: Difficulty finding the right word to say or expressing thoughts
Over the PAST 6 MONTHS, how MUCH has this symptom bothered you?: Difficulty finding the right word to say or expressing thoughts
27. Difficulty understanding things: Did you have this symptom BEFORE CFS?
Over the PAST 6 MONTHS, how OFTEN have you had this symptom?: Difficulty understanding things
Over the PAST 6 MONTHS, how MUCH has this symptom bothered you?: Difficulty understanding things
28. Only can focus on one thing at a time: Did you have this symptom BEFORE CFS?

Over the PAST 6 MONTHS, how OFTEN have you had this symptom?: Only can focus on one thing at a time
Over the PAST 6 MONTHS, how MUCH has this symptom bothered you?: Only can focus on one thing at a time
29. Unable to focus vision and attention: Did you have this symptom BEFORE CFS?
Over the PAST 6 MONTHS, how OFTEN have you had this symptom?: Unable to focus vision and attention
Over the PAST 6 MONTHS, how MUCH has this symptom bothered you?: Unable to focus vision and attention
30. Loss of depth perception: Did you have this symptom BEFORE CFS?
Over the PAST 6 MONTHS, how OFTEN have you had this symptom?: Loss of depth perception
Over the PAST 6 MONTHS, how MUCH has this symptom bothered you?: Loss of depth perception
31. Slowness of thought: Did you have this symptom BEFORE CFS?
Over the PAST 6 MONTHS, how OFTEN have you had this symptom?:Slowness of thought
Over the PAST 6 MONTHS, how MUCH has this symptom bothered you?:Slowness of thought
32. Absent-mindedness or forgetfulness: Did you have this symptom BEFORE CFS?
Over the PAST 6 MONTHS, how OFTEN have you had this symptom?: Absent-mindedness or forgetfulness
Over the PAST 6 MONTHS, how MUCH has this symptom bothered you?: Absent-mindedness or forgetfulness
33. Bladder problems: Did you have this symptom BEFORE CFS?
Over the PAST 6 MONTHS, how OFTEN have you had this symptom?: Bladder problems
Over the PAST 6 MONTHS, how MUCH has this symptom bothered you?: Bladder problems
34. Irritable bowel problems: Did you have this symptom BEFORE CFS?
Over the PAST 6 MONTHS, how OFTEN have you had this symptom?: Irritable bowel problems
Over the PAST 6 MONTHS, how MUCH has this symptom bothered you?: Irritable bowel problems
35. Nausea: Did you have this symptom BEFORE CFS?
Over the PAST 6 MONTHS, how OFTEN have you had this symptom?: Nausea
Over the PAST 6 MONTHS, how MUCH has this symptom bothered you?: Nausea
36. Feeling unsteady on your feet, like you might fall: Did you have this symptom BEFORE CFS?
Over the PAST 6 MONTHS, how OFTEN have you had this symptom?: Feeling unsteady on your feet, like you might fall
Over the PAST 6 MONTHS, how MUCH has this symptom bothered you?: Feeling unsteady on your feet, like you might fall
37. Shortness of breath or trouble catching your breath: Did you have this symptom BEFORE CFS?
Over the PAST 6 MONTHS, how OFTEN have you had this symptom?: Shortness of breath or trouble catching your breath
Over the PAST 6 MONTHS, how MUCH has this symptom bothered you?: Shortness of breath or trouble catching your breath
38. Dizziness or fainting: Did you have this symptom BEFORE CFS?
Over the PAST 6 MONTHS, how OFTEN have you had this symptom?: Dizziness or fainting
Over the PAST 6 MONTHS, how MUCH has this symptom bothered you?: Dizziness or fainting
39. Irregular heart beats: Did you have this symptom BEFORE CFS?
Over the PAST 6 MONTHS, how OFTEN have you had this symptom?: Irregular heart beats
Over the PAST 6 MONTHS, how MUCH has this symptom bothered you?: Irregular heart beats
40. Losing or gaining weight without trying: Did you have this symptom BEFORE CFS?
Over the PAST 6 MONTHS, how OFTEN have you had this symptom?: Losing or gaining weight without trying
Over the PAST 6 MONTHS, how MUCH has this symptom bothered you?: Losing or gaining weight without trying
41. No appetite: Did you have this symptom BEFORE CFS?
Over the PAST 6 MONTHS, how OFTEN have you had this symptom?: No appetite
Over the PAST 6 MONTHS, how MUCH has this symptom bothered you?: No appetite
42. Sweating hands: Did you have this symptom BEFORE CFS?
Over the PAST 6 MONTHS, how OFTEN have you had this symptom?: Sweating hands
Over the PAST 6 MONTHS, how MUCH has this symptom bothered you?: Sweating hands
43. Night sweats: Did you have this symptom BEFORE CFS?
Over the PAST 6 MONTHS, how OFTEN have you had this symptom?: Night sweats
Over the PAST 6 MONTHS, how MUCH has this symptom bothered you?: Night sweats
44. Cold limbs (e.g. arms, legs, hands): Did you have this symptom BEFORE CFS?
Over the PAST 6 MONTHS, how OFTEN have you had this symptom?: Cold limbs (e.g. arms, legs, hands)
Over the PAST 6 MONTHS, how MUCH has this symptom bothered you?: Cold limbs (e.g. arms, legs, hands)
45. Feeling chills or shivers: Did you have this symptom BEFORE CFS?
Over the PAST 6 MONTHS, how OFTEN have you had this symptom?: Feeling chills or shivers
Over the PAST 6 MONTHS, how MUCH has this symptom bothered you?: Feeling chills or shivers
46. Feeling hot or cold for no reason: Did you have this symptom BEFORE CFS?
Over the PAST 6 MONTHS, how OFTEN have you had this symptom?: Feeling hot or cold for no reason
Over the PAST 6 MONTHS, how MUCH has this symptom bothered you?: Feeling hot or cold for no reason
47. Feeling like you have a high temperature: Did you have this symptom BEFORE CFS?
Over the PAST 6 MONTHS, how OFTEN have you had this symptom?: Feeling like you have a high temperature
Over the PAST 6 MONTHS, how MUCH has this symptom bothered you?: Feeling like you have a high temperature
48. Feeling like you have a low temperature: Did you have this symptom BEFORE CFS?
Over the PAST 6 MONTHS, how OFTEN have you had this symptom?: Feeling like you have a low temperature
Over the PAST 6 MONTHS, how MUCH has this symptom bothered you?: Feeling like you have a low temperature
49. Alcohol intolerance: Did you have this symptom BEFORE CFS?
Over the PAST 6 MONTHS, how OFTEN have you had this symptom?: Alcohol intolerance
Over the PAST 6 MONTHS, how MUCH has this symptom bothered you?: Alcohol intolerance
50. Sore throat: Did you have this symptom BEFORE CFS?
Over the PAST 6 MONTHS, how OFTEN have you had this symptom?: Sore throat
Over the PAST 6 MONTHS, how MUCH has this symptom bothered you?: Sore throat
51. Tender/sore lymph nodes: Did you have this symptom BEFORE CFS?
Over the PAST 6 MONTHS, how OFTEN have you had this symptom?: Tender/sore lymph nodes
Over the PAST 6 MONTHS, how MUCH has this symptom bothered you?: Tender/sore lymph nodes
52. Fever: Did you have this symptom BEFORE CFS?
Over the PAST 6 MONTHS, how OFTEN have you had this symptom?: Fever
Over the PAST 6 MONTHS, how MUCH has this symptom bothered you?: Fever
53. Flu-like symptoms: Did you have this symptom BEFORE CFS?
Over the PAST 6 MONTHS, how OFTEN have you had this symptom?: Flu-like symptoms
Over the PAST 6 MONTHS, how MUCH has this symptom bothered you?: Flu-like symptoms
54. Some smells, foods, medications, or chemicals make you feel sick: Did you have this symptom BEFORE CFS?
Over the PAST 6 MONTHS, how OFTEN have you had this symptom? Some smells, foods, medications, or chemicals make you feel sick
Over the PAST 6 MONTHS, how MUCH has this symptom bothered you? Some smells, foods, medications, or chemicals make you feel sick

PSQI

1. During the past month, what time have you usually gone to bed at night?
2. During the past month, how long (in minutes) has it usually taken you to fall asleep each night?
3. During the past month, what time have you usually gotten up in the morning?
4. During the past month, how many hours of actual sleep did you get at night? (This may be different than the number of hours you spent in bed.)
5a) Cannot get to sleep within 30 minutes
5b) Wake up in the middle of the night or early morning
5c) Have to get up to use the bathroom
5d) Cannot breathe comfortably
5e) Cough or snore loudly
5f) Feel too cold
5g) Feel too hot
5h) Had bad dreams
5i) Have pain
5j) Other Reasons
5j) Other Reasons (Please specify)
6. During the past month, how would you rate your sleep quality overall?
7. During the past month, how often have you taken medicine to help you sleep (prescribed or over the counter)?
8. During the past month, how often have you had trouble staying awake while driving, eating meals, or engaging in social activity?
9. During the past month, how much of a problem has it been for you to keep up enough enthusiasm to get things done?
10. Do you have a bed partner or room mate?
10a) Loud snoring
10b) Long pauses between breaths while asleep
10c) Legs twitching or jerking while you sleep
10d) Episodes of disorientation or confusion during sleep
10e) Other restlessness while you sleep
10e) Other restlessness while you sleep; please describe

SF-36

1. In general, would you say your health is:
2. Compared to one year ago, how would your rate your health in general now?
3. Vigorous activities, such as running, lifting heavy objects, participating in strenuous sports
4. Moderate activities, such as moving a table, pushing a vacuum cleaner, bowling, or playing golf
5. Lifting or carrying groceries
6. Climbing several flights of stairs
7. Climbing one flight of stairs
8. Bending, kneeling, or stooping
9. Walking more than a mile
10. Walking several blocks
11. Walking one block
12. Bathing or dressing yourself
13. Cut down the amount of time you spent on work or other activities
14. Accomplished less than you would like
15. Were limited in the kind of work or other activities
16. Had difficulty performing the work or other activities (for example, it took extra effort)
17. Cut down the amount of time you spent on work or other activities
18. Accomplished less than you would like
19. Didnt do work or other activities as carefully as usual
20. During the past 4 weeks, to what extent has your physical health or emotional problems interfered with your normal social activities with family, friends, neighbors, or groups? (Choose one answer.)
21. How much bodily pain have you had during the past 4 weeks? (Choose one answer.)
22. During the past 4 weeks, how much did pain interfere with your normal work (including both work outside the home and housework)? (Choose one answer.)
23. Did you feel full of pep?
24. Have you been a very nervous person?
25. Have you felt so down in the dumps that nothing could cheer you up?
26. Have you felt calm and peaceful?
27. Did you have a lot of energy?
28. Have you felt downhearted and blue?
29. Did you feel worn out?
30. Have you been a happy person?
31. Did you feel tired?
32. During the past 4 weeks, how much of the time has your physical health or emotional problems interfered with your social activities (like visiting with friends, relatives, etc.)?(Choose one number)
33. I seem to get sick a little easier than other people
34. I am as healthy as anybody I know
35. I expect my health to get worse
36. My health is excellent

MFI

1. I feel fit.
2. Physically I feel only able to do a little.
3. I feel very active.
4. I feel like doing all sorts of nice things.
5. I feel tired.
6. I think I do a lot in a day.
7. When I am doing something, I can keep my thoughts on it.
8. Physically I can take on a lot.
9. I dread having to do things.
10. I think I do very little in a day.
11. I can concentrate well.
12. I am rested.
13. It takes a lot of effort to concentrate on things.
14. Physically I feel I am in a bad condition.
15. I have a lot of plans.
16. I tire easily.
17. I get little done.
18. I dont feel like doing anything.
19. My thoughts easily wander.
20. Physically I feel I am in an excellent condition.

Table S7.

A. ME/CFS vs. Control

Compound name	Case			Control		
	Median	25% quantile	75% quantile	Median	25% quantile	75% quantile
LPC 18:2	179492	155385	223064	248296	190397	351462
Betaine	1279930	1023228	1536118	1392119	1251990	1691588
TG 53:5	4928	3348	7020	3143	2126	4476
α N-phenylacetyl-L-glutamine	53165	30910	67632	30331	20633	48028
PC 30:0	76948	49105	130279	100156	74461	142271
SM d32:1	4753	3125	5747	5116	4056	5798
PC 33:0	5201	1213	7745	7183	5180	8368
Urobilin	4201	2687	9489	2861	1561	5375
ε Caprolactam	205675	181596	230038	199070	141933	210973
TG 54:8	1581	1012	3701	848	493	1417
γ Butyrobetaine	4493	1053	5513	5275	4342	6135
PC p34:2 or PC o34:3	54564	35879	67516	61816	54040	79247
TG 53:4	20915	15425	27333	14982	11319	21826
L-carnitine	174682	147927	198693	193258	168161	216571
TG 52:5	108366	70198	146070	68231	49268	103964
PC 28:0	1062	265	2493	1664	836	4030
PC 34:0	22338	19897	23905	24394	21452	26470
TG 54:6 A	97935	52860	218527	67738	39669	100803
PC 36:4 A	177344	121466	226481	206163	152152	273571
Indole-3-lactate	430	364	480	487	411	541
Tyr Met Lys	3453042	3032911	3810594	3134672	2783992	3512217
TG 52:4	1026752	772065	1361031	750095	550290	1067028
TG 52:3	2739390	2376790	3161104	2309462	1825497	2789254
PC 34:4	8274	4914	11888	9866	7878	12762
LPC 16:1	8670	6308	11088	10940	7778	12673
Acylcarnitine C9:1	3415	2638	4792	4010	2985	6353
PC 32:2	23469	19039	32235	30006	24087	37648
PC 32:0	114673	103817	130796	130088	115597	152724
PC p36:4 or PC o36:5	107071	83057	122311	112599	97049	144855
CE 18:3	2812	1281	3668	3253	2780	3956
PC 35:4	7225	5142	9828	9124	6901	11146
PC 34:1	2719857	2346822	3116607	3044490	2627182	3427536
TG 54:5 A	650126	433320	1139461	525607	351734	652639
DG 36:3	8317	5461	10930	6604	4630	8458
Acylcarnitine C11:1	5196	3074	8403	7477	4555	9700
PE p36:4 or PE o36:5	18904	14532	24565	22069	16799	27636

B. ME/CFS + IBS vs. Control

Compound name	Case w/ IBS			Control		
	Median	25% quantile	75% quantile	Median	25% quantile	75% quantile
LPC 18:2	172041	156986	193553	248296	190397	351462
Ceramide d36:1	6548	4225	8952	4925	3580	6077
γ Butyrobetaine	4493	1053	5513	5275	4342	6135
LPC 18:1	90932	82643	102614	121750	97896	173305
5-Methylthioadenosine	6761	5969	7562	5715	4937	6416
TG 49:2	23334	16527	39487	15809	10103	22368
Ceramide d40:0	10870	8716	11885	8137	6362	9878
TG 51:3	38843	32042	41581	27662	19003	35863
Betaine	1336374	970931	1496378	1392119	1251990	1691588
Ceramide d42:0	14066	8203	16400	9779	7643	12714
LPC p16:0 or LPC o16:1	3355	990	4478	4833	3583	5928
Ceramide d34:1	25249	18929	28429	21411	17553	24474
Indole-3-lactate	428	377	489	487	411	541
TG 50:3	374616	323258	483891	249998	175958	356389
TG 53:4	19253	16676	25733	14982	11319	21826
PE 38:6	56259	40084	72862	48664	38015	57611
TG 53:3	35998	28634	41843	29991	21995	36646
TG 51:4	8221	6596	11434	6073	4004	9118
TG 52:5	86571	69664	141503	68231	49268	103964
PC 36:4 A	159439	145519	215864	206163	152152	273571
TG 53:5	4375	3263	6087	3143	2126	4476
TG 51:2	58147	48054	73795	39693	27452	55735
TG 50:4	60581	38976	75780	36599	26522	56887
DG 34:1	2587	2219	3704	2067	1525	2890
Mannitol	2137	1577	3302	1735	1285	2034
PC 34:2	5470005	4849585	5905124	6014348	5273487	6848672
Ceramide d38:1	11234	8132	13010	8284	6251	9709
PC p32:0 or PC o32:1	11889	9966	14683	14718	10776	16925
PC p34:2 or PC o34:3	52116	35656	67870	61816	54040	79247
TG 51:1	8455	5491	20366	7723	3940	12688
TG 53:2	31992	23086	36417	25954	17009	31664
CE 18:1	20938	16688	26593	24321	19645	31587
PE 34:2	5258	4001	8043	3894	2669	6624
TG 52:4	818575	695793	1150735	750095	550290	1067028
L-alanine	65040	59436	74211	58825	45620	70459
L-carnitine	173333	158294	207513	193258	168161	216571
PE 38:4 B	48044	30210	53163	31741	25744	43455
TG 50:2	1102315	836462	1512512	908734	628674	1128742
PC 40:8	5842	4734	6814	6735	5517	7628
PE 36:4	17608	13963	29023	15550	10280	20660
Cholesterol	101990	85562	109649	109680	96311	130804
Ceramide d40:1	8156	6617	10783	6862	5353	10019
PE 36:2	13771	8986	16397	9752	7293	15240
TG 48:2	170124	109896	307751	109954	52831	193975

C. ME/CFS w/o IBS vs. Control						
Compound name	Case w/o IBS			Control		
	Median	25% quantile	75% quantile	Median	25% quantile	75% quantile
PC 33:0	3459	1055	5377	7183	5180	8368
PC 38:2	14995	12213	18071	17576	16323	19522
PC 30:0	69291	48294	93266	100156	74461	142271
tyrosine	20347	16466	23740	24568	20595	27234
PC 36:6	1893	629	4905	4060	3067	5772
TG 54:6 A	157556	85372	219191	67738	39669	100803
TG 53:5	5522	4101	7226	3143	2126	4476
TG 54:8	1955	1310	3945	848	493	1417
Tyr Met Lys	3500360	3216387	3642125	3134672	2783992	3512217
PC 32:2	22562	17031	28309	30006	24087	37648
PC 32:1	225118	171000	301427	297216	230582	433990
LPC 18:2	186390	151560	260684	248296	190397	351462
LPC 16:1	8485	5952	10386	10940	7778	12673
PC 32:1	7967	4121	13060	10048	7485	16660
SM d32:1	50537	38044	57484	56125	49836	68890
PC 28:0	537	178	1376	1664	836	4030
PC 34:4	6870	4699	9610	9866	7878	12762
Linoleic acid	1199	478	1567	647	419	1016
SM d32:2	4464	1606	5317	5116	4056	5798
PC 32:0	111191	102205	130538	130088	115597	152724
Acylcarnitine C9:1	3028	2609	4304	4010	2985	6353
TG 52:3	3005708	2489795	3400668	2309462	1825497	2789254
PC p34:2 or PC o34:3	57332	36185	67042	61816	54040	79247
Indole-3-lactate	431	343	476	487	411	541
PC 34:0	22279	19470	25076	24394	21452	26470
TG 54:4	1054750	819345	1483814	768365	550304	987215
TG 52:4	1116980	977290	1548461	750095	550290	1067028
TG 52:5	124159	76153	164000	68231	49268	103964
PC p36:4 or PC o36:5	93960	77738	116035	112599	97049	144855
DG 36:3	9336	5626	12295	6604	4630	8458
TG 14:0:14:0:14:0	2119	1454	4661	3215	2017	7794
PC 34:4	6870	4699	9610	9866	7878	12762
Urobilin	3873	3027	8379	2861	1561	5375
L-carnitine	176031	147802	191233	193258	168161	216571
PC 32:2	22562	17031	28309	30006	24087	37648
PC 32:0	111191	102205	130538	130088	115597	152724
PC 33:1	22967	16562	26167	28753	21858	33140
Betaine	1247715	1066878	1635242	1392119	1251990	1691588
PC 40:6 A	6474	4579	7777	7443	6483	9212
PC 37:3	5547	3677	6969	7326	5747	9323
Ceramide d43:1	4772	3155	8600	8535	5390	14003
Ceramide d42:1	21521	17220	27353	23923	20672	28491
PE p36:4 or PE o36:5	16869	12271	20816	22069	16799	27636
PC 36:4 A	184909	121143	228838	206163	152152	273571
PC 30:1	1162	633	1671	2037	1153	2837
PC 35:4	6824	5123	9664	9124	6901	11146
LPC 14:0	4905	3962	6663	6502	5333	8176
Acylcarnitine C11:1	5162	3005	7591	7477	4555	9700
PC 34:1	2627415	2293265	2881503	3044490	2627182	3427536
TG 53:4	22899	14951	29841	14982	11319	21826
CE 18:3	2799	1271	3186	3253	2780	3956
PC 38:2	14995	12213	18071	17576	16323	19522

D. ME/CFS vs. Control -female only						
Compound name	Case			Control		
	Median	25% quantile	75% quantile	Median	25% quantile	75% quantile
LPC 18:2	172820	148255	202626	240225	192702	322829
TG 53:5	4827	3604	6649	3086	2070	4073
TG 51:3	38843	26467	42954	26241	17324	32509
indole-3-lactate	414	354	468	496	415	541
LPC 18:2	34044	29468	50476	47279	40224	58203
TG 52:3	2726968	2386933	3101689	2215517	1748615	2585879
PC 30:0	76223	51538	119021	101328	78713	142772
γ-butyrobetaine	4161	1008	4934	5246	3839	6000
PC 28:0	838	222	2463.75	1664	836	4030
PC 33:0	5253	1571	7866	7356	5435	8595
LPC 20:2	1161.5	717	1373.75	1673	1170.5	2258.5
TG 52:5	106139	71800	144185	63321	47856	87770
LPC 18:1	90790	77781	106761	121099	97600	170828
TG 52:4	1041107	802408	1339656	691854	547075	989282
LPE 18:2	2317	443	4406	4528	646	6526
L-carnitine	166322	140578	188782	192733	167381	211060
TG 53:3	37010	27672	44800	27965	21952	32993
CE 18:3	2805	1201.5	3702	3317	2831	3969
SM d32:1	51650	43801	67176	57182	50917	69434
PC 32:0	115152	105669	130615	132739	120687	155446
PC 34:0	22498	20035	24060	25367	21762	27109
TG 51:4	8258	6596	13168	5719	3758	8261
PC 40:8	6060	4787	6814	6797	5838	7681
TG 53:4	19477	15703	27294	13891	9657	18879
TG 54:5 A	634811	445412	1138307	485318	345512	649238
leucine	34134	29909	39505	37018	31813	45228
PC p-36:4 or PC o-36:5	108159	82672	125831	113840	97776	147665
DG 36:3	8490	5470	10623	6425	4570	8095
TG 54:6 A	87674	54708	197908	63453	37117	98049
LPC 16:1	8378	6455	10943	11069	7795	12737
PC p-34:2 or PC o-34:3	56321	36185	67053	63667	54736	83434
PC 35:4	7411	5700	9846	9571	6926	11525
PC 34:1	2689627	2338224	3129772	3095847	2654918	3424823
PC p-34:2 or PC o-34:3	8394	7171	10719	11966	8132	13633
LPC 16:0	670902	598646	798955	826534	625583	930460
Acylcarnitine C9:1	3438.5	2668	4528	4279	2968	6354
LPC 18:1	24750	20596	33925	32757	24877	46558
PC p-36:4 or PC o-36:5	8025	5725	10892	9997	7372	12330
PE p-36:4 or PE o-36:5	19005	15183	26547	24180	17264	28425
